# Genome-wide identification of the *RPD3/HDA1* gene family in foxtail millet (*Setaria italica*) and analysis of their association with plant height

**DOI:** 10.3389/fpls.2025.1722313

**Published:** 2026-01-05

**Authors:** Hongkai Liang, Zhangfei Zheng, Puyuan Yang, Xiaokuo Cui, Liwei Wang, Hui Zhi, Guanqing Jia, Qiang He, Xianmin Diao

**Affiliations:** 1 School of Tropical Agriculture and Forestry, Hainan University, Haikou, China; 2 State Key Laboratory of Crop Gene Resources and Breeding, Institute of Crop Sciences, Chinese Academy of Agricultural Sciences, Beijing, China; 3 College of Agronomy, Hebei Agricultural University, Baoding, China

**Keywords:** *Setaria italica*, RPD3/HDA1, histone deacetylase, plant height, haplotype analysis, phytohormone response

## Abstract

The *RPD3/HDA1* histone deacetylase family members play crucial roles in plant development, influencing traits such as plant height, stress response and fertility. Although, the *RPD3/HDA1* family has been characterized in several plant species, its members and functions in foxtail millet remain largely unknown. In this study, we identified 13 *SiRPD3/HDA1* genes in foxtail millet and classified them into four distinct phylogenetic groups. Structural analysis revealed conserved exon-intron patterns and motif compositions within each group, implying functional conservation. Two segmental duplication events were detected. The Ka/Ks ratios (<1) suggested that these gene pairs have undergone strong purifying selection. Expression profiling showed that most *SiRPD3/HDA1* genes were expressed during reproductive stages, particularly in shoot apical meristem. Promoter analysis revealed multiple phytohormone-responsive *cis*-elements. Consistently, qRT-PCR assays confirmed that exogenous GA, ABA, MeJA, and IAA triggered distinct, gene-specific expression responses, together suggesting functional divergence of *SiRPD3/HDA1* genes in hormone signaling. Importantly, we performed haplotype-based association analysis across 942 natural accessions, covering the entire *SiRPD3/HDA1* gene family. This analysis revealed extensive natural variations and identified five candidate genes (*Seita.1G034500*, *Seita.2G042600*, *Seita.4G235100*, *Seita.5G190100*, and *Seita.5G255900*) showing strong association with plant height. Subsequently, subcellular localization analysis showed that Seita.2G042600 and Seita.5G190100 are targeted to the nucleus and plasma membrane, respectively, suggesting their distinct functional roles. This study provides a comprehensive characterization of the *RPD3/HDA1* family in foxtail millet, identifies potential regulators associated with plant height, and lays a foundation for future functional studies and molecular breeding efforts.

## 1 Introduction

Histone acetylation is one of the most common epigenetic modifications in plants and is dynamically regulated by two opposing enzyme families. Histone acetyltransferases (HATs), add acetyl groups to loosen chromatin, thereby activating transcription. In contrast, histone deacetylases (HDACs) remove acetyl groups, promoting chromatin condensation and repressing gene repression (Kuo and Allis, 1998; Pandey et al., 2002). This antagonistic balance maintains acetylation homeostasis and enables rapid transcriptional reprogramming during plant growth and environmental adaptation (Chen and Tian, 2007). HDACs are highly conserved across eukaryotes and act as key chromatin-remodeling regulators through the deacetylation of histone N-terminal tails (Tahir and Tian, 2021). In plants, HDACs are categorized into three families: *RPD3/HDA1* family (reduced potassium dependency 3/histone deacetylase 1), the *HD2* (histone deacetylase 2) family, and the SIR2 (silent information regulator 2) family (Yang and Seto, 2007; Bourque et al., 2016). Among them, the *RPD3/HDA1* family is not only the largest but also most functionally diverse. Its name was derived from sequence similarity to the yeast proteins Hda1 and Rpd3, and members of this family possess zinc-dependent catalytic activity.

In plants, members of the *RPD3/HDA1* family have been shown to play key roles in diverse biological processes, including seed germination, flowering time, plant height, fertility, hormone signaling, and stress responses (Ma et al., 2013). This family has been investigated in many plant species, such as Arabidopsis, rice, maize, cotton and buckwheat, with gene numbers ranging from 8 to 18 (Pandey et al., 2002; Chu and Chen, 2018; Zhang et al., 2020a; Zhang et al., 2020b; Hou et al., 2021, Hou et al., 2022). Functional analyses in crops highlight their developmental importance. In maize, overexpression of *ZmHDA101* resulted in reduced growth and delayed flowering (Rossi et al., 2007). In rice, RNAi silencing of *OsHDA703* leads to shortened peduncles, decreased fertility and altered flowering time and vegetative growth (Hu et al., 2009; Wang et al., 2020). Consistently, RNAi and knockout of Os*HDA704* and Os*HDA714* also reduced plant height (Hu et al., 2009; Xu et al., 2021). In addition, several rice *RPD3/HDA1* genes exhibit hormone-responsive expression: *OsHDA705* is induced by jasmonic acid (JA), abscisic acid (ABA) and biotic stresses, while *OsHDA702* is upregulated by salicylic acid (SA), JA, and ABA (Fu et al., 2007; Zhao et al., 2016). In *Arabidopsis*, the *RPD3/HDA1* family is divided into three subclasses, Class I (AtHDA6, AtHDA7, AtHDA9 and AtHDA19), Class II (AtHDA5, AtHDA14, AtHDA15 and AtHDA18), Class III (AtHDA2) and Class IV (AtHDA8) (Fu et al., 2007). Among them, *AtHDA19* is a key regulator of global transcriptional and its suppression leads to multiple developmental defects (Fong et al., 2006; Tian and Chen, 2001). It also controls root cortex cell expansion and hormone signaling pathways (Wakeel et al., 2018; Chen et al., 2019). *AtHDA15* interacts with *AtPIF1* to repress seed germination, whereas *AtHDA6* regulates flowering time, seed development, circadian rhythm control and transposon silencing (Wu et al., 2008; Chhun et al., 2016; Gu et al., 2017; Hung et al., 2019; Yang et al., 2020). Together, these findings underscore the functional diversity and biological significance of *RPD3/HDA1* family members in plant growth and development.

Foxtail millet (*Setaria italica*) is an important cereal crop widely cultivated in East Asia, characterized by its self-fertilization, drought tolerance and broad adaptability. It has gained increasing attention as a model C_4_ plant due to its short reproductive cycle, compact diploid genome and suitability for functional genomics (Doust et al., 2009; Diao et al., 2014). Although members of the *RPD3/HDA1* histone deacetylase family have been functionally characterized in several plant species, their roles in foxtail millet remain largely unexplored. In particular, plant height—a key agronomic trait affecting biomass accumulation, lodging resistance, and overall yield—has not yet been genetically linked to any HDAC family members in this species. To address this gap, we conducted a genome-wide identification of the *SiRPD3/HDA1* gene family in foxtail millet, and investigated their evolutionary relationships, gene structures, conserved motifs, and expression profiles. Furthermore, by integrating haplotype-based association mapping and hormone-induced expression assays, we identified candidate genes potentially associated with plant height regulation. These findings provide a valuable foundation for understanding the epigenetic regulation of plant architecture and for guiding molecular breeding strategies in foxtail millet.

## 2 Materials and methods

### 2.1 Genome-wide identification of *RPD3/HDA1* genes in foxtail millet

To identify candidate *RPD3/HDA1* gene members, Hidden Markov Model (HMM) files (PF0085) were downloaded from the Pfam database (https://pfam.xfam.org/) (El-Gebali et al., 2019). Protein sequence files for foxtail millet (JGI_V2.2), *Sorghum bicolor* (L.) Moench (JGI_V3.1.1), *Oryza sativa* L.(JGI_V7.0), *Zea mays* L.(JGI_V4), *Brachypodium distachyon* (L.) Beauv.(JGI_V3.2) and *Hordeum vulgare* L.(JGI_Vr1) were retrieved from the JGI database (https://phytozome-next.jgi.doe.gov/) (Goodstein et al., 2012). Identification was performed using HMMER 3.0 software with an E-value threshold of 1e-10 (Finn et al., 2011). Structural domain validation was subsequently performed using the SMART (http://smart.embl.de/) and NCBI conserved domain databases (https://www.ncbi.nlm.nih.gov/cdd/), with sequences containing the Hist_deacetyl domain being classified as *RPD3/HDA1* family members. Additionally, physicochemical properties including amino acid length, molecular weight, isoelectric point, hydrophilicity as well as subcellular localization were determined using ExPASy (http://web.expasy.org/protparam/) and Softberry (http://www.softberry.com/berry.phtml?topic=protcompan&group=programs&subgroup=proloc).

### 2.2 Phylogenetic and syntenic analysis of the *SiRPD3* gene family

Protein sequences of *SiRPD3* genes from six species were aligned using ClustalW implemented in MEGA 7.0 software under default alignment parameters (Kumar et al., 2016). A neighbor-joining (NJ) phylogenetic tree was constructed with 1000 bootstrap replications and subsequently refined using the iTOL (https://itol.embl.de/) (Letunic and Bork, 2021). The classification of SiRPD3/HDA1 genes was determined based on the phylogenetic tree topology and bootstrap support values, following the classification systems reported for RPD3/HDA1 families in rice (Fu et al., 2007), Arabidopsis (Hollender and Liu, 2008), and cotton (Zhang et al., 2020). Gene duplication events involving *SiRPD3* genes were identified using the MCScanX toolkit with default parameters (Wang et al., 2012). Additionally, substitution rates for non-synonymous (Ka), and synonymous (Ks) mutations, as well as their ratio (Ka/Ks), were calculated among different gene pairs, using the Nei-Gojobori (NG) method implemented in TBtools (Chen et al., 2020).

### 2.3 Chromosomal distribution, gene structure, conserved motif, and *cis*-acting element prediction analysis of the *SiRPD3/HDA1* gene family

Chromosomal location information for *SiRPD3* genes was extracted from the foxtail millet genome annotation file (GFF3) and their distribution was visualized using Mapchart software (Voorrips, 2002). Exon-intron structures were illustrated by importing the corresponding GFF3 file into TBtools (Chen et al., 2020). Conserved motifs in *SiRPD3* proteins were analyzed using the MEME 5.5.2 online tool (https://meme-suite.org/meme/tools/meme) with the Maximum number of motifs set to 10 and the results were subsequently visualized using TBtools (Chen et al., 2020). Additionally, the 2000 bp upstream sequences of *SiRPD3* genes were analyzed for *cis*-regulatory elements using the PlantCARE database (http://bioinformatics.psb.ugent.be/webtools/plantcare/html/).

### 2.4 Protein interaction network of *SiPRD3/HDA1* genes

The protein interaction network of the SiRPD3 was analyzed using the STRING database (https://cn.string-db.org/), with *Arabidopsis* homologs serving as references. The resulting network was then visualized and refined using Cytoscape software (V3.10.0) (Shannon et al., 2003).

### 2.5 Expression patterns of *SiRPD3/HDA1* gene family

The expression pattern of *SiRPD3* genes were analyzed using transcriptomic data in Setaria-db website (http://www.setariadb.com/millet) which were derived from tissues of the foxtail millet variety Yugu1, collected at different developmental stages under standard laboratory conditions (He et al., 2023b). Hierarchical clustering of *SiRPD3* genes expression levels, based on FPKM values, was performed using the heatmap function in TBtools.

### 2.6 Haplotype analysis of the *SiRPD3/HDA1* family numbers

All single nucleotide polymorphisms (SNPs) in *SiRPD3* genes were obtained from high-depth resequencing data of 942 foxtail millet accessions (He et al., 2023a, He et al., 2023b). The SNP data were filtered using a python script to retain only variable sites within the coding sequences (CDS) of *SiRPD3* members. Additionally, plant height phenotypic data for 680 core germplasm resources were retrieved from the *Setaria-*db database (He et al., 2023b). Haplotype identification and the subsequent association analysis between *SiRPD3* haplotypes and plant height phenotypes were performed using geneHapR (Zhang et al., 2023). The statistical significance of phenotypic differences among haplotypes was evaluated using one-way analysis of variance (ANOVA) followed by Tukey’s honestly significant difference (HSD) test (*p* < 0.05).

### 2.7 Plant material cultivation and treatment

In this experiment, the foxtail millet variety Yugu1 was used as plant material. Seeds were sown in pots and cultivated in a greenhouse at the Institute of Crop Science, Chinese Academy of Agricultural Sciences (CAAS), Beijing, China. Plants were grown under controlled conditions with a 10-hour light/14-hour dark photoperiod at 28°C. For gene expression analysis using reverse Transcription-quantitative polymerase chain reaction (RT-qPCR), tissue samples were collected from seedlings at the five-leaf stage. Seedlings were divided into two groups: a control group (CK-grown) and a treatment group sprayed with 100 µM solutions of gibberellic acid (GA), abscisic acid (ABA), methyl jasmonate (MeJA), or indole-3-acetic acid (IAA), respectively (Nan et al., 2024). Samples were harvested at 0, 1, 6, 12, and 24 hours after treatment, immediately frozen in liquid nitrogen and stored at −80°C. Three biological replicates were collected for each treatment and time point.

### 2.8 RNA extraction and RT-qPCR expression analysis

Total RNA was extracted from Yugu1 seedings following the protocol described by He et al (He et al., 2023b). First-strand cDNA was synthesized using the All-In-One Ultra RT Super Mix for qPCR kit (Vazyme Biotechnology Co., Ltd., China). RT-PCR was performed using the 2x Realtime PCR Super Mix SYBR Green with anti-Taq Mix kit (Mei5 Biotechnology Co., Ltd.). Gene-specific primers were designed using Beacon Designer 8 software and are listed in **Supplementary Table S4**. The *Setaria italica* β-actin gene was used as the internal reference. Relative expression levels were calculated using the 2^−ΔΔCT^ method. Line plots of gene expression profiles were generated using the ggplot2 package in R (version 4.3.2). Statistical analyses were conducted using one-way ANOVA, and multiple comparisons among groups were performed with Tukey’s HSD test (*p* < 0.05).

### 2.9 Subcellular localization assays

RNA isolation and cDNA synthesis were performed as previously described (He et al., 2023b). The coding sequences of *Seita.2G042600* and *Seita*.*5G190100*, excluding the stop codon, were amplified by PCR and subsequently fused in-frame with GFP in the transient expression vector *pCAMBIA1305Ubi-GFP* (Invitrogen). The resulting constructs, designated as *pUbi::Seita.2G042600-GFP* and *pUbi::Seita.5G190100-GFP*, were introduced into *Agrobacterium tumefaciens* strain EHA105. For transient expression assays, the recombinant *Agrobacterium* strains were co-infiltrated with the P19 (pSoup-p19) into *Nicotiana benthamiana* leaves using a mixed infiltration method. Subcellular localization of the GFP fusion proteins was examined in *N. benthamiana* epidermal cells 48–60 h post-infiltration (hpi) using a confocal laser scanning microscope (LSM700, Zeiss, Germany). The nuclei and plasma membrane (PM) were stained with DAPI and FM4-64, respectively, and bright-field imaging was used to provide morphological context. Primers used in this study are listed in **Supplementary Table S5**.

## 3 Results

### 3.1 Genome-wide identification of *RPD3/HDA1* genes in six species and analysis of *SiRPD3/HDA1* family

Hist_deacetyl (PF00850) domain based analysis identified a total of 76 *RPD3/HDA1* genes were identified across six species. Among these, 11 genes were found in (*Brachypodium distachyon* (L.) Beauv.), 11 in (*Sorghum bicolor* (L.) Moench), 14 in (*Oryza sativa* L.), 13 in (*Hordeum vulgare* L.), and 14 in (*Zea mays* L.), and 13 *SiRPD3* genes in foxtail millet (**Supplementary Table S1**). Seita.2G381100 was the largest, with 771 amino acids (aa), whereas Seita.3G130200 was the smallest with 309 aa. Molecular weights ranged from 32.62 kDa (Seita.3G130200) to 83.95 kDa (Seita.2G381100) average 51.34 kDa. The pI values varied between 4.98 (Seita.7G088900) and 10.23 (Seita.1G034500) with an average value of 6.15. GRAVY scores ranged from -0.606 to -0.053, indicating that SiRPD3/HDA1 proteins are hydrophilic. The SiRPD3/HDA1 proteins were predicted to localize to multiple subcellular compartments. Specifically, four were associated with the chloroplast, five with the nucleus, and five with the cytoplasm, suggesting diverse regulatory roles for this protein family (**Supplementary Table S1**).

### 3.2 Phylogenetic relationship and gene duplication analysis of the *RPD3/HDA1* gene family

**Figure 1:**
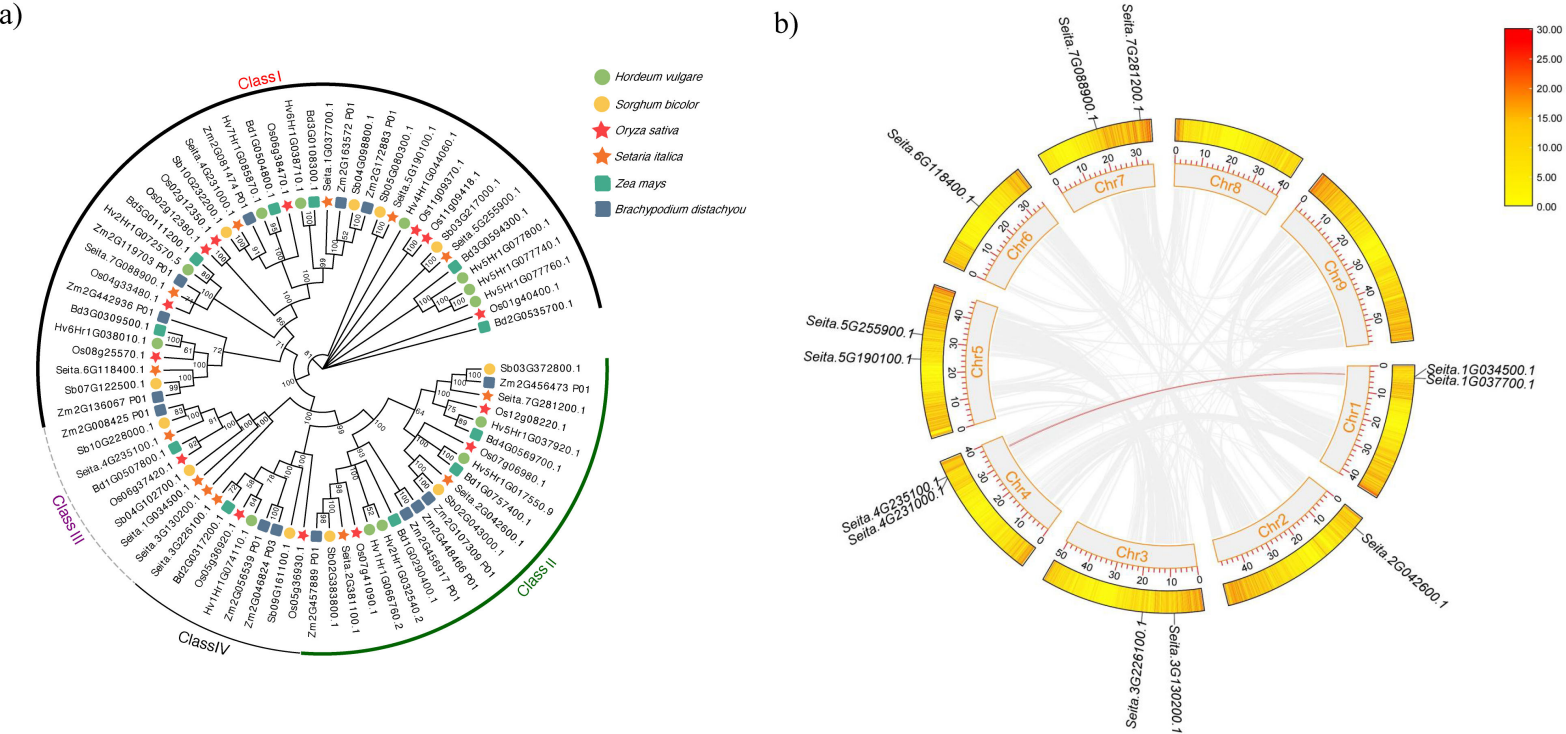
Phylogenetic and synteny analysis of the *RPD3/HDA1* gene family. **(a)** The neighbor-joining phylogenetic tree of *RPD3/HDA1* proteins from six plant species. Multiple sequences alignment and phylogenetic tree were constructed by MEGA7.0 and the bootstrap test was performed with 1000 iterations. The different colored shapes represent different species, and the four subgroups are distinguished by four types of lines; **(b)** Synteny analysis of *SiPRD3/HDA1* genes. The gray areas indicate all synteny blocks in foxtail millet genome and orange blocks indicate foxtail millet chromosomes (Chr1-9). The red lines indicate segment duplicated *SiPRD3/HDA1* gene pairs.

To investigate the phylogenetic relationships of the *SiRPD3/HDA1* genes among different species, a phylogenetic tree was constructed based on 76 RPD3/HDA1 protein sequences (**Figure 1a**). As shown in **Figure 1a**, the RPD3/HDA1 proteins were classified into four groups: Class I, Class II, Class III, and Class IV. Class I was the largest subgroup, comprising 39 members, while Class III was the smallest, containing only 8 members: 3 from *SiRPD3*, 2 from *SbRPD3*, and 1 each from *ZmRPD3*, *OsRPD3* and *BdRPD3.* Notably, no *HvRPD3* protein clustered within Class III. However, *RPD3/HDA1* genes from Class I, Class II, and the Class IV group included *RPD3/HDA1* genes from all six species, indicating that the *RPD3/HDA1* family is relatively conserved throughout the evolution history of these species.

**Figure 2:**
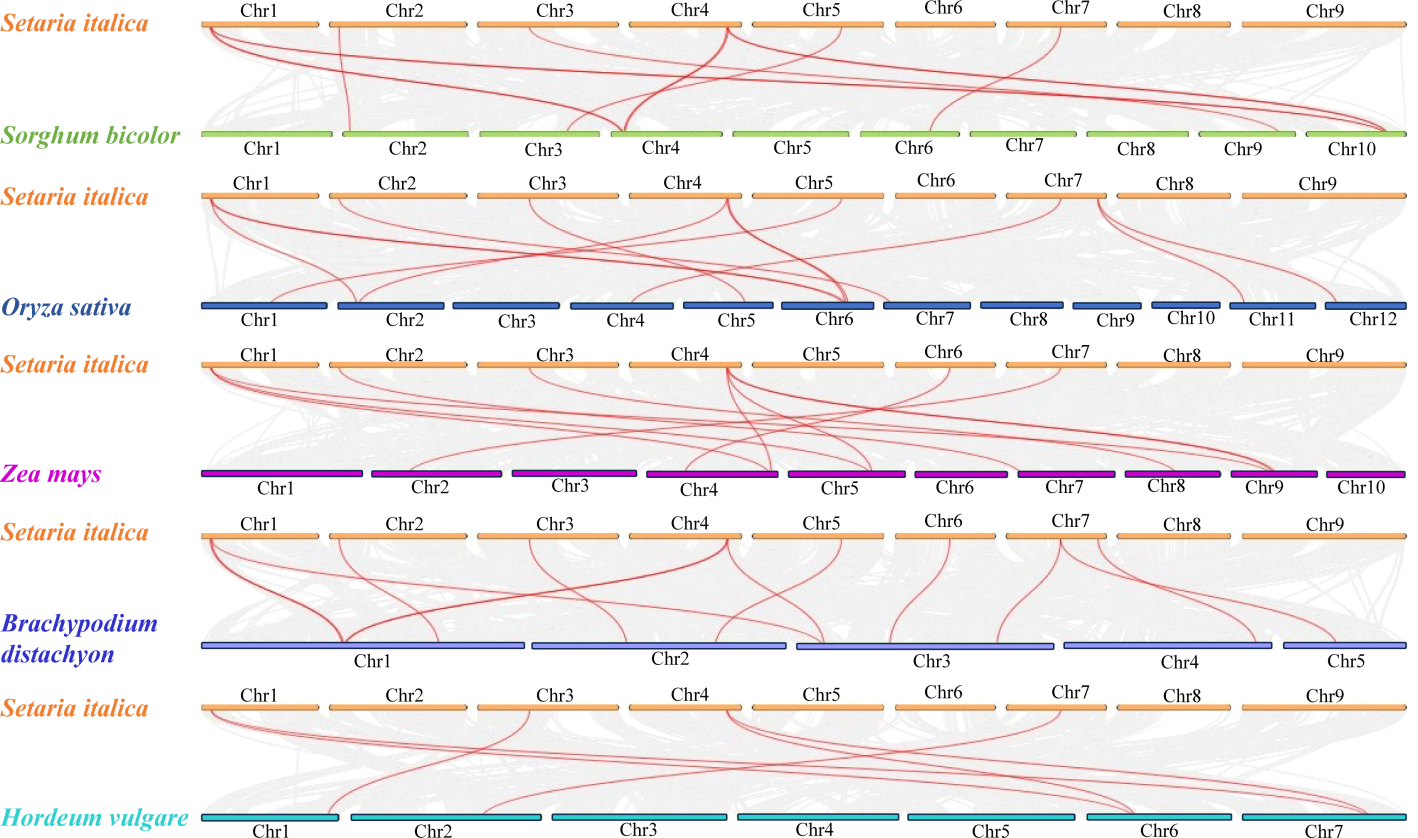
Synteny analysis of *PRD3* genes between foxtail millet and *Sorghum bicolor Moench, Oryza sativa, Zea mays, Brachypodium distachyon* and *Hordeum vulgare*, respectively. Gray lines in the background indicate the collinear blocks within foxtail millet and other plant genomes, while red lines indicate the orthologous relationship of *PRD3/HDA1* genes.

On further analysis of expansion and evolution of *SiPRD3* genes, two pairs of segmental duplication events were identified: *Seita.1G034500*/*Seita.4G235100* and *Seita.1G037700*/*Seita.4G231000*, suggesting possible functional diversification via subfunctionalization or neofunctionalization (**Figure 1b**; **Supplementary Table S2**). Syntenic analysis between foxtail millet and five representative monocots species, (*B. distachyon*, *S.bicolor*, *O. sativa*, *H. vulgare*, and *Z. mays*) revealed species-specific conservation patterns: with *B. distachyon* having the highest number of syntenic gene pairs (13), followed by *O. sativa* (12), *S.bicolor* (12) and *Z. mays* (11), while *H. vulgare* exhibited the lowest conservation (6) (**Figure 2****,****Supplementary Table S3**). Furthermore, analysis of Ka/Ks ratios showed values consistently below 1 for the segmental duplication gene pairs, indicating that the *PRD3/HDA1* gene family in foxtail millet has experienced strong purifying selection pressure throughout its evolution history (**Supplementary Table S2**).

### 3.3 Chromosomal distribution, gene structure and motif composition of *SiRPD3/HDA1* gene family

**Figure 3:**
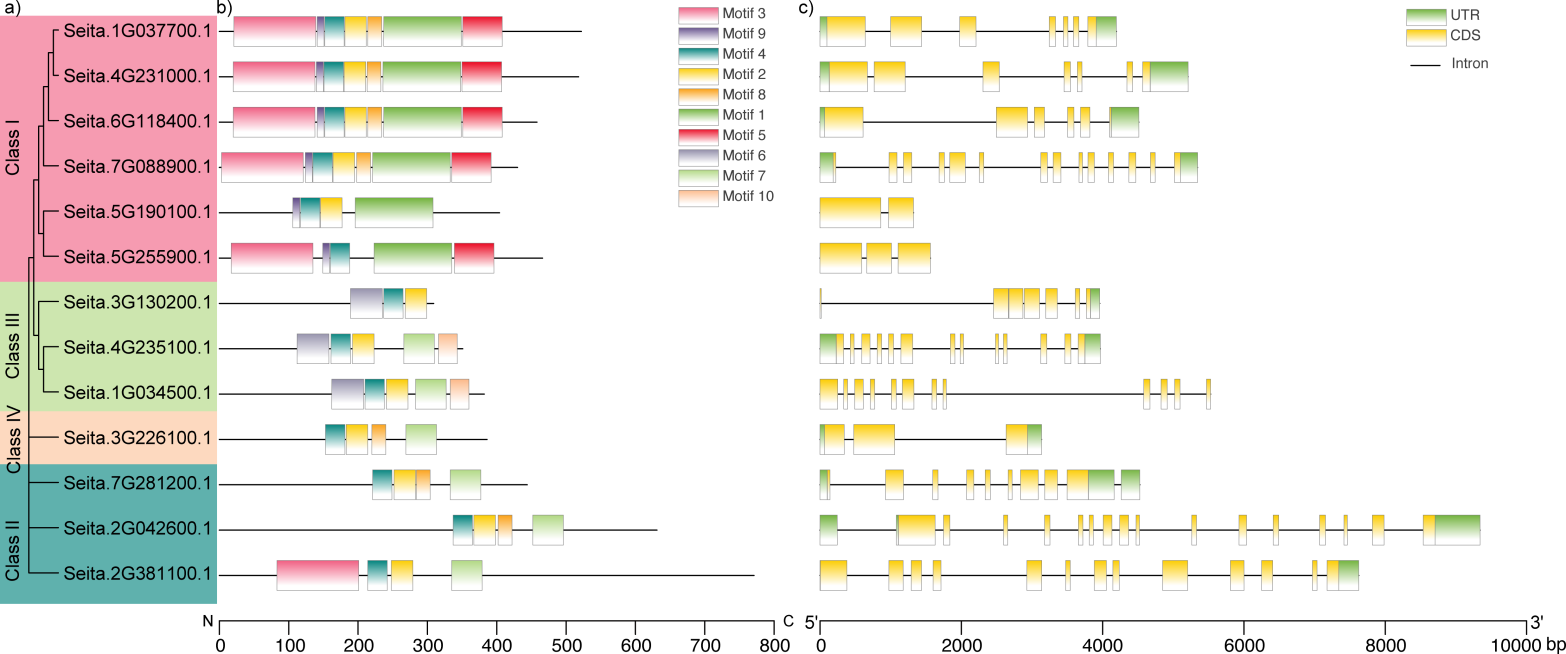
Phylogenetic tree, conserved motif and gene structure of the *RPD3/HDA1* gene family of foxtail millet. **(a)** A neighbor-joining phylogenetic tree of 13 foxtail millet *RPD3/HDA1* members, classified into four subgroups and distinguished by four different colored backgrounds; **(b)** Conserved motifs of 13 foxtail millet RPD3 members; **(c)** Gene structure of 13 foxtail millet *RPD3/HDA1* members. UTR, exons, and introns, indicated by green box, yellow box and black line, respectively.

The similarities and differences among the *SiRPD3/HDA1* genes we were analyzed by a phylogenetic analysis and comparison of conserved motifs and gene structure (**Figure 3**). The phylogenetic tree grouped the 13 SiPRD3/HDA1 protein sequences into four classes (**Figure 3a**). Class I is the largest, comprising 6 members, while Class II and Class III each contain 3 members and with one gene remaining Class IV. Motif analysis revealed that *SiRPD3/HDA1* genes within the same subgroup shared highly similar motif compositions, consistent with their phylogenetic relationships (**Figures 3a, b**). Most Class I members contained seven conserved motifs, while Class II and III members generally harbored four to five motifs. Notably, motif 4 was present in all proteins, suggesting its essential role in maintaining the catalytic activity of the conserved Hist_deacetyl domain. In contrast, subgroup-specific variations in motif organization may reflect functional diversification among family members. Interestingly, although the Class IV member (*Seita.3G226100*) clusters separately from Class II, their motif architectures are partially similar, suggesting retention of certain conserved structural features despite evolutionary divergence. Gene structure analysis supported these findings, as genes within the same subgroup displayed comparable exon–intron architectures, with exon numbers ranging from 3 to 16 (**Figure 3c**). Most *SiRPD3/HDA1* genes contained both upstream and downstream regions adjacent to the coding sequence (CDS), except *Seita.5G190100*, *Seita.5G255900*, *Seita.1G034500*, and *Seita.2G042600*, which displayed truncated or missing flanking regions (**Figure 3c**).

### 3.4 Promoter analysis and protein interaction prediction of *SiRPD3/HDA1*

**Figure 4:**
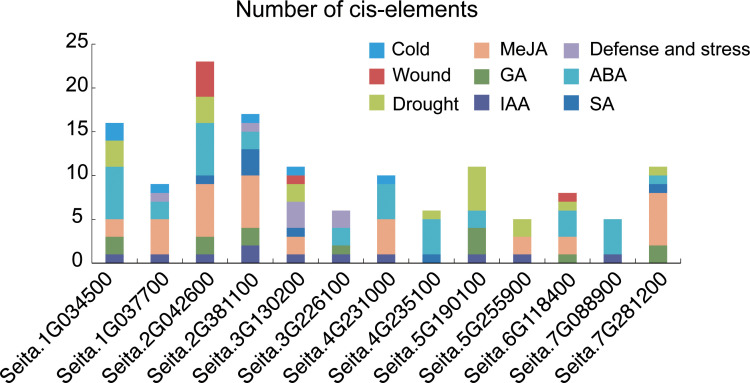
Analysis of cis-acting elements of promoters of *SiRPD3/HDA1* family members. The different colors represent the different cis-acting elements.

Potential functions and transcriptional regulatory mechanisms of *SiRPD3* genes were explored by prediction *cis*-acting elements within the 2000 bp promoter regions upstream of the 13 *SiRPD3* genes. Analysis at PlantCARE database identified a diverse array of *cis*-acting elements associated with plant hormone responses, environmental stress responses, and plant growth and development (**Figure 4**). A total of nine elements related to plant hormones and four stress responses elements were identified. Promoter analysis revealed that most SiRPD3/HDA1 genes contained multiple hormone-responsive cis-elements, predominantly associated with ABA, MeJA, and IAA signaling pathways (**Figure 4**). Among them, ABA-responsive elements (ABREs) were the most abundant (detected in 11 of 13 genes), followed by MeJA- (CGTCA/TGACG motifs) and IAA-responsive (TGA-elements) elements. These results suggest that SiRPD3/HDA1 genes may play important roles in multiple phytohormone signaling pathways, particularly ABA and jasmonate responses.

To further predict potential interacting proteins of SiRPD3/HDA1, we constructed a protein interaction network using orthologous Arabidopsis proteins from the STRING database and visualized the results with Cytoscape. A total of ten functional proteins were identified as directly or indirectly associated with SiRPD3/HDA1 proteins (**Supplementary Figure S1**). These include development-related proteins (HOS15), stress response proteins (WRKY, PRH, and HAT3.1), protein trafficking factors (EREL1/2 and EREX), chromatin remodeling factors (MRG1/2), and histone deacetylation regulators (SNL2). These findings suggest that several SiRPD3/HDA1 proteins may play important roles in plant development, stress responses, and chromatin remodeling.

### 3.5 Expression patterns of *SiRPD3/HDA1* genes in different tissues and developmental stages

**Figure 5:**
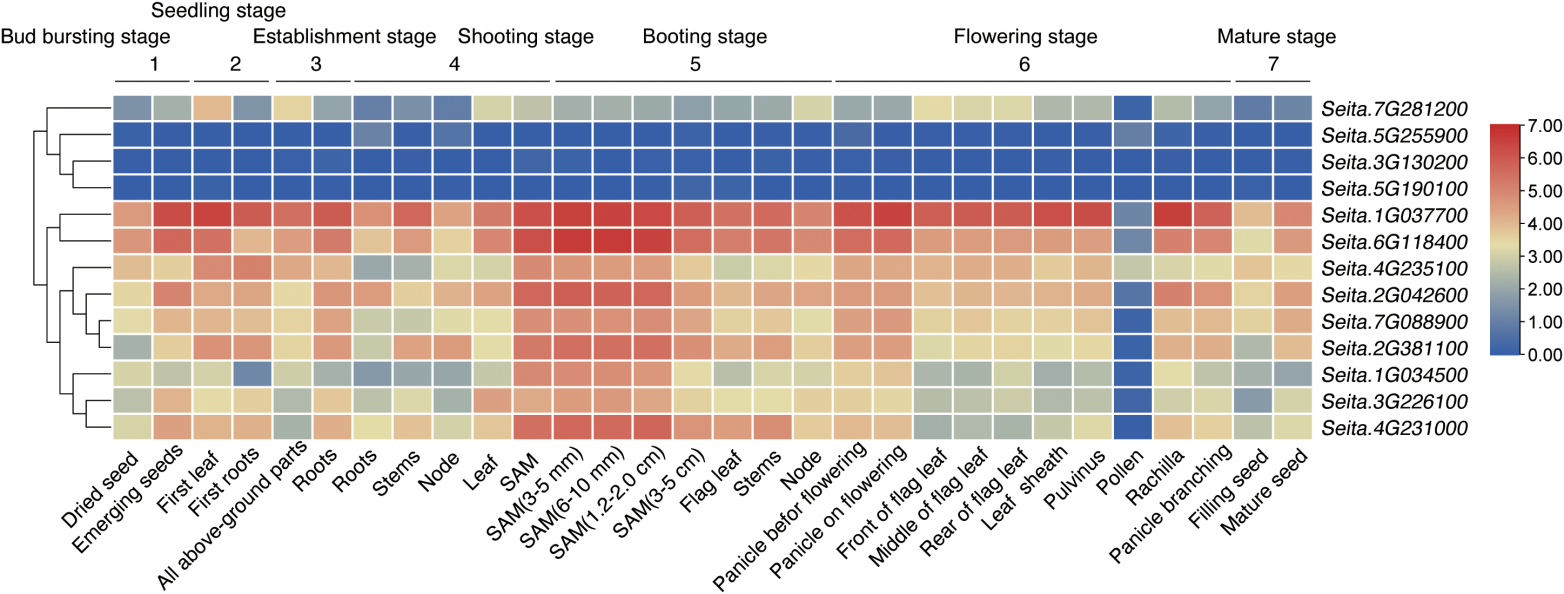
Expression pattern of *SiRPD3/HDA1* under normal conditions in foxtail millet. Expression patterns of 13 *RPD3/HDA1* genes in different tissues at seven stages in foxtail millet. The log2 (FPKM+1) values are showed in boxes.

Analysis of expression patterns of *SiRPD3/HDA1* genes using transcriptome data from various tissues throughout the reproductive period revealed diverse expression patterns, with FPKM values ranging widely from 0 to 101.4 (**Figure 5**). Nine genes (*Seita.1G034500, Seita.1G037700, Seita.2G042600, Seita.2G381100 Seita.3G226100, Seita.4G231000, Seita.4G235100, Seita.6G118400*, and *Seita.7G088900*) showed high expression in multiple tissues, particularly in the shoot apical meristem (SAM), suggesting important roles in SAM development. In contrast, all *SiRPD3/HDA1* genes exhibited minimal expressed in pollen. Additionally, *Seita.1G037700*, *Seita.2G381100*, *Seita.2G042600*, *Seita.4G231000*, and *Seita.6G118400* showed high expression levels in stems or nodes during the shooting and booting stages. Conversely, three genes (*Seita.5G255900, Seita.3G130200*, and *Seita.5G190100*) had consistently low expression throughout the reproductive stages, indicating potential functional differentiation within the *SiRPD3/HDA1* family.

### 3.6 Haplotype analyses of the *SiRPD3/HDA1* family genes

**Figure 6:**
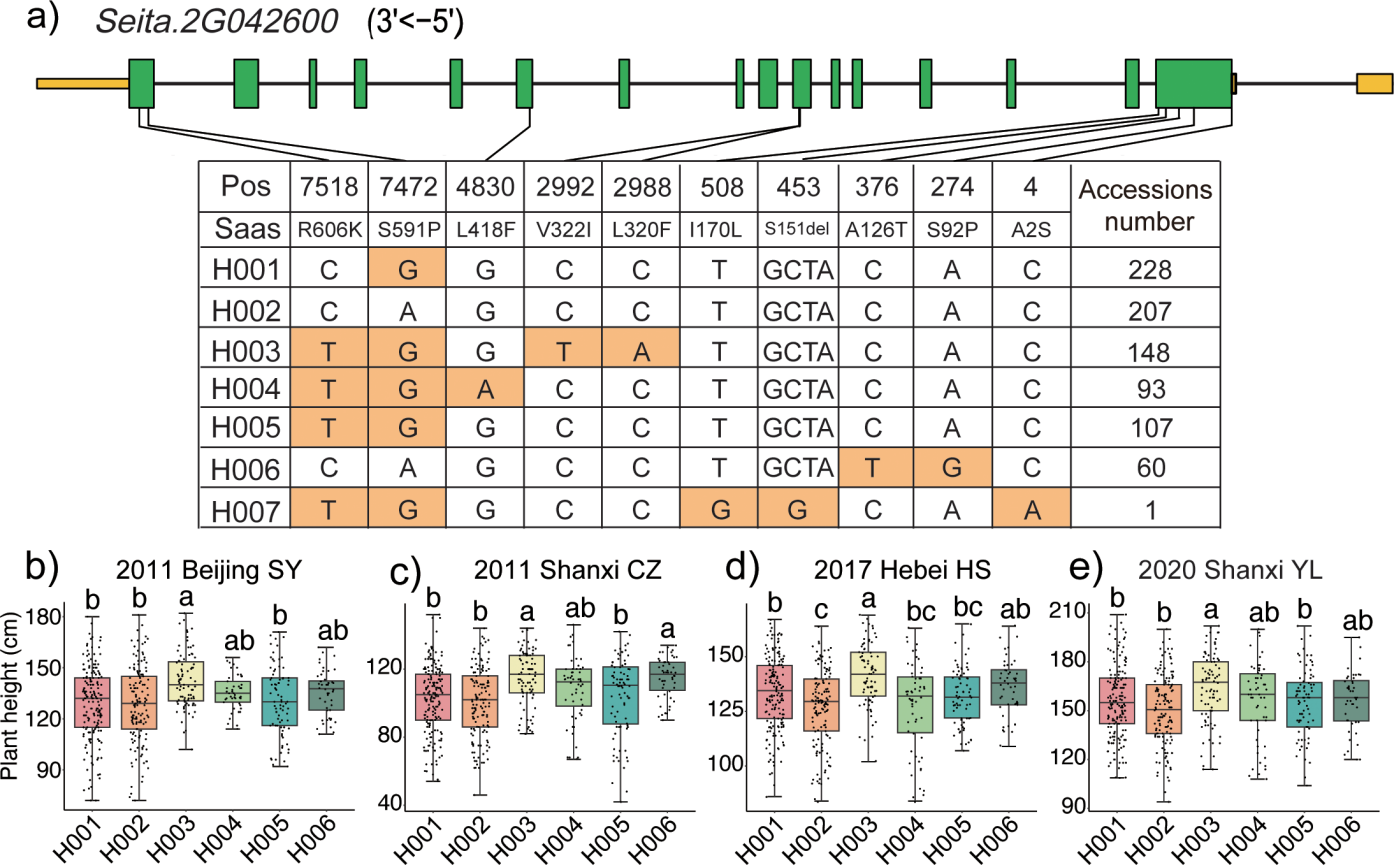
Haplotype analysis of *Seita.2G042600* in exons to assess effects on foxtail millet plant height. **(a)** UTRs, exons, and introns are indicated by yellow boxes, green boxes and black lines, respectively, with orange indicating base changes. Saas: the single amino acid substitution; Accessions number: number of materials possessed by each haplotype; **(b–e)** Plant height of *Seita.2G042600* haplotypes evaluated across four geographical sites: Shunyi, Beijing (SY); Changzhi, Shanxi Province (CZ); Hengshui, Hebei Province (HS); and Yulin, Shaanxi Province (YL). Different lowercase letters indicate significant differences (Tukey’s HSD, P < 0.05). Each dot represents one sample.

Haplotype analysis of the 13 *SiRPD3/HDA1* genes was done using 942 core-collected foxtail millet accessions to investigate the correlation between variation *SiRPD3/HDA1* and plant height. Detailed results for all 13 genes, including haplotype classification, accession lists, and corresponding plant height values, are provided in **Supplementary Tables S5**–**S13**. Nine genes have non-synonymous mutant SNPs ranging from 1 to 10, with the remaining four genes having no non-synonymous mutations (**Figure 6**; **Supplementary Figures S2**–**S9**). The homologue of *Seita.2G042600* is *HDA704* (*LOC_Os07g06980)* in rice, which has been shown to cause rice mutants to exhibit a dwarf or semi-dwarf phenotype (Hu et al., 2009). For *Seita.2G042600*, there were nine SNPs and one indel in the CDS region that caused amino acid substitutions, resulting in the identification of seven haplotypes (**Figure 6a**). Hap2 is consistent with the allele of the reference genome Yugu1. In SY, CZ and YL H003 was significantly higher than H001, H002, H005 (*P* < 0.05); in HS H003 was significantly higher than H001, H002, H004, H005 (**Figures 6c–e**; **Supplementary Table S14**). H003 exhibits a high plant height phenotype, but this trait may be influenced by the environments.

For *Seita.1G034500*, there were two SNPs in the CDS region that caused amino acid substitution, and there was a significant difference in plant height between the two haplotypes identified (**Supplementary Figure S2a**). Under all four environments, accessions with Hap2 exhibited a significantly reduced plant height compared to accessions with Hap1 (*P* < 0.05) (**Supplementary Figures S2b–e**). The results suggest that Hap2 may contribute to the genetic improvement of semi-dwarf varieties in foxtail millet. Two SNPs causing amino acid substitution and one splice region variant type were identified in *Seita.4G235100*, resulting in four haplotypes (**Supplementary Figure S3a**). In the four environments, H002 exhibited significantly reduced plant height compared to other haplotypes (**Supplementary Figures S3b–e**). For *Seita.5G190100*, five SNPs and one Indels were detected, defining three haplotypes with H003 characterized as the dwarfing haplotype. The only notable difference between the H003 and H001 was at position 30, where a 7 bp insertion occurred (**Supplementary Figure S4**). Three principal haplotypes of *Seita.5G255900* were identified, determined by seven SNPs in the coding region (**Supplementary Figure S5a**). In all environments H003 exhibited significantly reduced plant height compared to H001 and H002 (*P* < 0.01), making H003 the favorable haplotype for breeding short-stature varieties (**Supplementary Figures S5b–e**).

For *Seita.3G226100*, *Seita.7G088900*, and *Seita.7G281200*, three, two and three haplotypes were identified, respectively (**Supplementary Figures S6**–**S8**). The haplotypes and plant height of these genes were analyzed for association, but the results did not show a significant difference among each haplotype. *Seita.2G381100* was identified with two haplotypes, but statistical was not possible for H002 as it was represented by only two sample the material (**Supplementary Figure S9a**). No loci with non-synonymous mutations were detected in the remaining *Seita.1G037700*, *Seita.3G130200*, *Seita.4G231000*, and *Seita.6G118400* genes (**Supplementary Figures S9b–e**). These results suggest that the *Seita.1G034500*, *Seita.2G042600*, *Seita.4G235100*, *Seita.5G190100*, and *Seita.5G255900* genes may play a role in plant height in foxtail millet.

### 3.7 Phytohormonal responses and cellular localization of key *SiRPD3/HDA1* genes

To explore the functional relevance of SiRPD3/HDA1 genes in plant height regulation, we selected five candidate genes (*Seita.2G042600*, *Seita.1G034500*, *Seita.4G235100*, *Seita.5G190100*, and *Seita.5G255900*) based on haplotype-phenotype association analyses. Given the well-established roles of gibberellin (GA), abscisic acid (ABA), methyl jasmonate (MeJA), and indole-3-acetic acid (IAA) in plant height regulation, we investigated the transcriptional responsiveness of these genes to exogenous hormone treatments.

**Figure 7:**
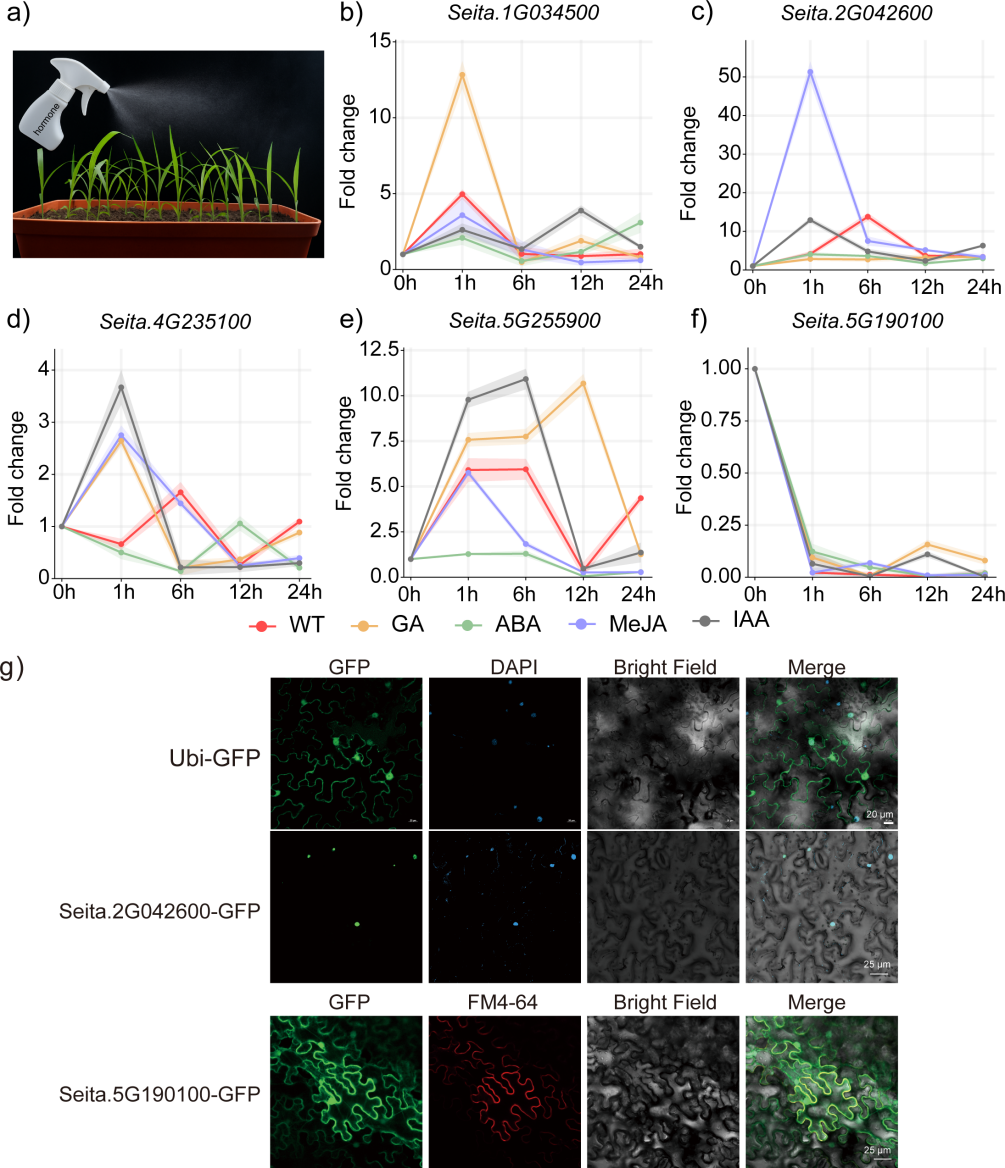
Phytohormone responsiveness and subcellular localization of SiRPD3/HDA1 candidate genes in foxtail millet. **(a)** Schematic illustration of hormone treatment on foxtail millet seedlings. Plants were treated with gibberellin (GA), abscisic acid (ABA), methyl jasmonate (MeJA), and indole-3-acetic acid (IAA), and sampled at 0, 1, 6, 12, and 24 hours post-treatment. **(b–f)** qRT-PCR analysis of the relative expression levels of five candidate SiRPD3/HDA1 genes under different hormone treatments: **(b)***Seita.1G034500*, **(c)***Seita.2G042600*, **(d)***Seita.4G235100*, **(e)***Seita.5G255900*, and **(f)***Seita.5G190100*. WT denotes untreated control. Each line represents a different hormone treatment, with shaded areas indicating standard deviations from three biological replicates. **(g)** Subcellular localization of *Seita.2G042600* and *Seita.5G190100*. GFP-tagged fusion proteins were transiently expressed in Nicotiana benthamiana epidermal cells. DAPI was used to stain nuclei (blue), and FM4-64 labeled plasma membranes (red). Merged images show GFP fluorescence overlaid with corresponding organelle markers and bright-field views. Seita.2G042600-GFP localized primarily to the nucleus, while Seita.5G190100-GFP showed plasma membrane localization.

Yugu1 seedlings were treated with 100 μM GA, ABA, MeJA, or IAA, and RT-qPCR was performed at 0, 1, 6, 12, and 24 h post-treatment to assess expression dynamics. The five SiRPD3/HDA1 family genes exhibited distinct and hormone-specific transcriptional responses (**Figures 7a–f**; **Supplementary Table S15**). Under GA treatment, *Seita.1G034500* and *Seita.4G235100* showed rapid and strong early induction, both peaking at 1 h, whereas *Seita.5G255900* and *Seita.5G190100* displayed sustained upregulation with maximal expression at 12 h. In contrast, *Seita.2G042600* was consistently downregulated across all time points following GA exposure. ABA treatment led to strong repression of gene expression, particularly for *Seita.5G255900* and *Seita.2G042600*, which showed marked reductions at early stages (1–6 h), followed by a gradual recovery at later time points. Under MeJA treatment, *Seita.2G042600* exhibited an exceptionally sharp and transient induction at 1 h (>50-fold), followed by a rapid decline, whereas *Seita.1G034500*, *Seita.4G235100*, and *Seita.5G255900* showed moderate early induction and decreased thereafter. Under IAA treatment, *Seita.2G042600* and *Seita.4G235100* showed a rapid and transient induction, reaching their highest expression at 1 h and declining thereafter. In contrast, *Seita.1G034500* and *Seita.5G190100* exhibited delayed induction with peaks at 12 h, whereas *Seita.5G255900* increased steadily from 1 h to 6 h, reached a maximum at 6 h, and then decreased at 24 h. To further support their functional roles, subcellular localization analysis was performed for *Seita.2G042600* and *Seita.5G190100* (**Figure 7g**). As shown in **Figure 7g**, the GFP signal of *Seita.2G042600*-GFP strongly co-localization with DAPI in the nucleus, indicating nuclear targeting, whereas *Seita.5G190100*-GFP merged well with FM4-64, suggesting that it is localized at the PM.

Collectively, our findings reveal that the five candidate *SiRPD3/HDA1* genes are differentially responsive to plant height-related hormones. Moreover, subcellular localization of two representative genes points to functional divergence at the cellular level, reinforcing their potential involvement in hormone-mediated growth regulation.

## 4 Discussion

Histone deacetylases (HDACs) are essential epigenetic regulators that modulate chromatin structure and broadly influence plant growth, development, and stress responses (Ruijter et al., 2003; Ma et al., 2013; Wang et al., 2014). Within this superfamily, the *RPD3/HDA1* subgroup is the most conserved and the most studied. In this study, we comprehensively identified 13 *SiRPD3/HDA1* genes in foxtail millet and classified them into four subfamilies based on phylogenetic relationships supported by bootstrap values and by reference to *RPD3/HDA1* classifications reported in rice (Fu et al., 2007), Arabidopsis (Hollender and Liu, 2008), and cotton (Zhang et al., 2020). Members within the same subfamily exhibited highly conserved exon–intron structures and motif organizations, whereas pronounced structural divergence was observed among subfamilies, consistent with patterns in other plant species (Pandey et al., 2002; Hou et al., 2022). These findings suggest that the *SiRPD3/HDA1* gene family has undergone both evolutionary conservation and functional diversification in foxtail millet.

Haplotype–phenotype association is a powerful approach for identifying natural variants linked to agronomically important traits (Liang et al., 2023; Zhang et al., 2023). Among the 13 *SiRPD3/HDA1* genes, *Seita.2G042600* and *Seita.1G034500* showed significant association with plant height variation across multiple environments. The functional relevance of *Seita.2G042600* is supported by studies of its rice homolog *OsHDA704*, whose knockdown leads to reduced plant height (Hu et al., 2009). *Seita.2G042600* also exhibits strong expression in the shoot apical meristem and is localized to the nucleus, consistent with its putative role in chromatin-mediated regulation of stem elongation. For *Seita.1G034500*, the dwarfing haplotype (H002) appears to have originated from the reference haplotype (H001). Although its rice homolog *OsHDA706* induces transient dwarfism when overexpressed, plants later recover normal height, suggesting a complex regulatory mechanism (Yang et al., 2024). The low expression of *Seita.1G034500* in Yugu1 stems and nodes further implies that its role in height regulation may occur during specific developmental windows. Functional validation—such as targeted knockouts or overexpression—will be required to clarify its regulatory mechanism in foxtail millet.

Three additional genes—*Seita.4G235100*, *Seita.5G190100*, and *Seita.5G255900*—also displayed haplotype associations with plant height. Although their expression levels in stems and nodes were generally low — with *Seita.4G235100* showing slightly higher basal expression than the other two (**Figure 5**), — the haplotypes carrying the mutant variants consistently corresponded to reduced plant height across multiple environments. This pattern suggests that these genes may influence plant height through upstream regulatory pathways or context-dependent mechanisms rather than through strong expression in elongating internodes. Interestingly, the distinct subcellular localization of Seita.5G190100 to the plasma membrane, in contrast to the nuclear localization of Seita.2G042600, implies mechanistic divergence. Nuclear localization aligns with classical HDAC-mediated transcriptional regulation, whereas the plasma membrane association of Seita.5G190100 may indicate involvement in upstream signaling or protein–protein interactions prior to nuclear response. However, the mechanistic connection between membrane localization and height regulation remains to be elucidated and warrants future investigation.

Promoter and hormone-response analyses further support functional divergence among *SiRPD3/HDA1* genes (**Figure 7**). The presence of abundant ABA-, MeJA-, and IAA-responsive cis-elements, together with gene-specific induction or repression under hormone treatments, indicates that members of this family participate in distinct phytohormone signaling pathways. The strong and transient MeJA response of *Seita.2G042600*, the GA/IAA dual responsiveness of *Seita.5G255900*, and the universal repression of *Seita.5G190100* by all four hormones underscore their diverse transcriptional regulatory behaviors. These differences, combined with their distinct expression profiles and subcellular localizations, highlight the potential functional specialization of *SiRPD3/HDA1* genes in growth regulation.

Taken together, our findings identify five *SiRPD3/HDA1* genes as promising candidates involved in natural variation in plant height. However, we emphasize that haplotype associations, expression profiles, and subcellular localization provide correlative—not causal—evidence. Given the global regulatory nature of HDACs, it is likely that *SiRPD3/HDA1* genes act within broader chromatin and hormonal regulatory networks rather than as isolated determinants of plant height. Future work integrating GWAS, transcriptomics, chromatin accessibility profiling, and functional genomics (e.g., CRISPR/Cas9 or overexpression studies) will be essential to define the specific roles of *SiRPD3/HDA1* genes and to position them within the height-regulatory network of foxtail millet.

## Data Availability

The original contributions presented in the study are included in the article/**Supplementary Material**. Further inquiries can be directed to the corresponding author.

## Supplementary material

Supplementary Table 1 Physicochemical parameters of 13 SiRPD3/HDA1 genes in *Setaria italica*

Supplementary Table 2 Segmentally duplicated *SiPRD3/HDA1* gene pairs and Ka, Ks, and Ka/Ks ratio

Supplementary Table 3 Syntenic PRD3/HDA1 gene pairs between foxtail millet and *Sorghum bicolor Moench*, *Oryza sativa*, *Zea mays*, *Brachypodium distachyon* and *Hordeum vulgare*.

Supplementary Table 4 Primers sequences used in this study

Supplementary Table 5 Plant Height Variation of Foxtail Millet Accessions Grouped by Seita.2G042600 Haplotypes

Supplementary Table 6 Plant Height Variation of Foxtail Millet Accessions Grouped by Seita.1G034500 Haplotypes

Supplementary Table 7 Plant Height Variation of Foxtail Millet Accessions Grouped by Seita.3G226100 Haplotypes

Supplementary Table 8 Plant Height Variation of Foxtail Millet Accessions Grouped by Seita.4G235100 Haplotypes

Supplementary Table 9 Plant Height Variation of Foxtail Millet Accessions Grouped by Seita.5G190100 Haplotypes

Supplementary Table 10 Plant Height Variation of Foxtail Millet Accessions Grouped by Seita.5G255900 Haplotypes

Supplementary Table 11 Plant Height Variation of Foxtail Millet Accessions Grouped by Seita.7G088900 Haplotypes

Supplementary Table 12 Plant Height Variation of Foxtail Millet Accessions Grouped by Seita.7G281200 Haplotypes

Supplementary Table 13 Plant Height Variation of Foxtail Millet Accessions Grouped by Seita.7G281100 Haplotypes

Supplementary Table 14 Statistical analysis of plant height significance among haplotypes of different genes.

Supplementary Table 15 Statistical analysis of differential gene expression among hormone treatments and time points

Supplementary Figure 1 SiPRD3/HDA1 homologous protein interaction network in *Arabidopsis*.

Supplementary Figure 2 Haplotype analysis of *Seita.1G034500* in exons to assess effects on foxtail millet plant height

Supplementary Figure 3 Haplotype analysis of *Seita.4G235100* in exons to assess effects on foxtail millet plant height.

Supplementary Figure 4 Haplotype analysis of *Seita.5G190100* in exons to assess effects on foxtail millet plant height.

Supplementary Figure 5 Haplotype analysis of *Seita.5G255900* in exons to assess effects on foxtail millet plant height.

Supplementary Figure 6 Haplotype analysis of *Seita.3G226100* in exons to assess effects on foxtail millet plant height.

Supplementary Figure 7 Haplotype analysis of *Seita.7G088900* in exons to assess effects on foxtail millet plant height.

Supplementary Figure 8 Haplotype analysis of *Seita.7G281200* in exons to assess effects on foxtail millet plant height.

Supplementary Figure 9 Haplotype analysis of *Seita.2G381100, Seita.1G037700, Seita.3G130200, Seita.4G231000* and *Seita.6G118400* in exons to assess effects on foxtail millet plant height.

## References

[B1] Bourque S. Jeandroz S. Grandperret V. Lehotai N. Aime S. Soltis D. E. . (2016). The evolution of HD2 proteins in green plants. Trends Plant Sci. 21, 1008–1016. doi: 10.1016/j.tplants.2016.10.001, PMID: 27789157

[B2] Chen C. Chen H. Zhang Y. Thomas H. R. Frank M. H. He Y. . (2020). TBtools: an integrative toolkit developed for interactive analyses of big biological data. Mol. Plant 13, 1194–1202. doi: 10.1016/j.molp.2020.06.009, PMID: 32585190

[B3] Chen W.-Q. Drapek C. Li D.-X. Xu Z.-H. Benfey P. N. Bai S.-N. (2019). Histone deacetylase HDA19 affects root cortical cell fate by interacting with SCARECROW. Plant Physiol. 180, 276–288. doi: 10.1104/pp.19.00056, PMID: 30737268 PMC6501111

[B4] Chen Z. J. Tian L. (2007). Roles of dynamic and reversible histone acetylation in plant development and polyploidy. Biochim. Biophys. Acta (BBA)-Gene Structure Expression 1769, 295–307. doi: 10.1016/j.bbaexp.2007.04.007, PMID: 17556080 PMC1950723

[B5] Chhun T. Chong S. Y. Park B. S. Wong E. C. C. Yin J.-L. Kim M. . (2016). HSI2 repressor recruits MED13 and HDA6 to down-regulate seed maturation gene expression directly during Arabidopsis early seedling growth. Plant Cell Physiol. 57, 1689–1706. doi: 10.1093/pcp/pcw095, PMID: 27335347

[B6] Chu J. Chen Z. (2018). Molecular identification of histone acetyltransferases and deacetylases in lower plant Marchantia polymorpha. Plant Physiol. Biochem. 132, 612–622. doi: 10.1016/j.plaphy.2018.10.012, PMID: 30336381

[B7] Diao X. Schnable J. Bennetzen J. L. Li J. (2014). Initiation of Setaria as a model plant. Front. Agric. Sci. Eng. 1, 16–20. doi: 10.15302/J-FASE-2014011

[B8] Doust A. N. Kellogg E. A. Devos K. M. Bennetzen J. L. (2009). Foxtail millet: a sequence-driven grass model system. Plant Physiol. 149, 137–141. doi: 10.1104/pp.108.129627, PMID: 19126705 PMC2613750

[B9] El-Gebali S. Mistry J. Bateman A. Eddy S. R. Luciani A. Potter S. C. . (2019). The Pfam protein families database in 2019. Nucleic Acids Res. 47, D427–D432. doi: 10.1093/nar/gky995, PMID: 30357350 PMC6324024

[B10] Finn R. D. Clements J. Eddy S. R. (2011). HMMER web server: interactive sequence similarity searching. Nucleic Acids Res. 39, W29–W37. doi: 10.1093/nar/gkr367, PMID: 21593126 PMC3125773

[B11] Fong P. M. Tian L. Chen Z. J. (2006). Arabidopsis thaliana histone deacetylase 1 (AtHD1) is localized in euchromatic regions and demonstrates histone deacetylase activity in *vitro*. Cell Res. 16, 479–488. doi: 10.1038/sj.cr.7310059, PMID: 16699543 PMC1986662

[B12] Fu W. Wu K. Duan J. (2007). Sequence and expression analysis of histone deacetylases in rice. Biochem. Biophys. Res. Commun. 356, 843–850. doi: 10.1016/j.bbrc.2007.03.010, PMID: 17399684

[B13] Goodstein D. M. Shu S. Howson R. Neupane R. Hayes R. D. Fazo J. . (2012). Phytozome: a comparative platform for green plant genomics. Nucleic Acids Res. 40, D1178–D1186. doi: 10.1093/nar/gkr944, PMID: 22110026 PMC3245001

[B14] Gu D. Chen C.-Y. Zhao M. Zhao L. Duan X. Duan J. . (2017). Identification of HDA15-PIF1 as a key repression module directing the transcriptional network of seed germination in the dark. Nucleic Acids Res. 45, 7137–7150. doi: 10.1093/nar/gkx283, PMID: 28444370 PMC5499575

[B15] He Q. Tang S. Zhi H. Chen J. Zhang J. Liang H. . (2023a). A graph-based genome and pan-genome variation of the model plant Setaria. Nat. Genet. 55, 1232–1242. doi: 10.1038/s41588-023-01423-w, PMID: 37291196 PMC10335933

[B16] He Q. Wang C. Zhang J. Liang H. Lu Z. Xie K. . (2023b). A complete reference genome assembly for foxtail millet and Setaria-db, a comprehensive database for Setaria. Mol. Plant S1674-2052, (1623) 00411–00412., PMID: 38155573 10.1016/j.molp.2023.12.017

[B17] Hollender C. Liu Z. (2008). Histone deacetylase genes in Arabidopsis development. J. Integr. Plant Biol. 50, 875–885. doi: 10.1111/j.1744-7909.2008.00704.x, PMID: 18713398

[B18] Hou J. Ren R. Xiao H. Chen Z. Yu J. Zhang H. . (2021). Characteristic and evolution of HAT and HDAC genes in Gramineae genomes and their expression analysis under diverse stress in Oryza sativa. Planta 253, 1–22. doi: 10.1007/s00425-021-03589-1, PMID: 33606144

[B19] Hou Y. Lu Q. Su J. Jin X. Jia C. An L. . (2022). Genome-Wide Analysis of the HDAC Gene Family and Its Functional Characterization at Low Temperatures in Tartary Buckwheat (Fagopyrum tataricum). Int. J. Mol. Sci. 23, 7622. doi: 10.3390/ijms23147622, PMID: 35886971 PMC9319316

[B20] Hu Y. Qin F. Huang L. Sun Q. Li C. Zhao Y. . (2009). Rice histone deacetylase genes display specific expression patterns and developmental functions. Biochem. Biophys. Res. Commun. 388, 266–271. doi: 10.1016/j.bbrc.2009.07.162, PMID: 19664599

[B21] Hung F.-Y. Chen F.-F. Li C. Chen C. Chen J.-H. Cui Y. . (2019). The LDL1/2-HDA6 histone modification complex interacts with TOC1 and regulates the core circadian clock components in Arabidopsis. Front. Plant Sci. 10, 233. doi: 10.3389/fpls.2019.00233, PMID: 30863422 PMC6399392

[B22] Kumar S. Stecher G. Tamura K. (2016). MEGA7: molecular evolutionary genetics analysis version 7.0 for bigger datasets. Mol. Biol. Evol. 33, 1870–1874. doi: 10.1093/molbev/msw054, PMID: 27004904 PMC8210823

[B23] Kuo M. H. Allis C. D. (1998). Roles of histone acetyltransferases and deacetylases in gene regulation. Bioessays 20, 615–626. doi: 10.1002/(SICI)1521-1878(199808)20:8<615::AID-BIES4>3.0.CO;2-H 9780836

[B24] Letunic I. Bork P. (2021). Interactive Tree Of Life (iTOL) v5: an online tool for phylogenetic tree display and annotation. Nucleic Acids Res. 49, W293–W296. doi: 10.1093/nar/gkab301, PMID: 33885785 PMC8265157

[B25] Liang H. He Q. Zhang H. Zhi H. Tang S. Wang H. . (2023). Identification and haplotype analysis of SiCHLI: a gene for yellow–green seedling as morphological marker to accelerate foxtail millet (Setaria italica) hybrid breeding. Theor. Appl. Genet. 136, 24. doi: 10.1007/s00122-023-04309-x, PMID: 36739566

[B26] Ma X. Lv S. Zhang C. Yang C. (2013). Histone deacetylases and their functions in plants. Plant Cell Rep. 32, 465–478. doi: 10.1007/s00299-013-1393-6, PMID: 23408190

[B27] Nan L. Li Y. Ma C. Meng X. Han Y. Li H. . (2024). Identification and expression analysis of the WOX transcription factor family in foxtail millet (Setaria italica L.). Genes 15, 476. doi: 10.3390/genes15040476, PMID: 38674410 PMC11050393

[B28] Pandey R. MuÈller A. Napoli C. A. Selinger D. A. Pikaard C. S. Richards E. J. . (2002). Analysis of histone acetyltransferase and histone deacetylase families of Arabidopsis thaliana suggests functional diversification of chromatin modification among multicellular eukaryotes. Nucleic Acids Res. 30, 5036–5055. doi: 10.1093/nar/gkf660, PMID: 12466527 PMC137973

[B29] Rossi V. Locatelli S. Varotto S. Donn G. Pirona R. Henderson D. A. . (2007). Maize histone deacetylase hda101 is involved in plant development, gene transcription, and sequence-specific modulation of histone modification of genes and repeats. Plant Cell 19, 1145–1162. doi: 10.1105/tpc.106.042549, PMID: 17468264 PMC1913744

[B30] Ruijter A. J. D. Gennip A. H. V. Caron H. N. Kemp S. Kuilenburg A. B. V. (2003). Histone deacetylases (HDACs): characterization of the classical HDAC family. Biochem. J. 370, 737–749. doi: 10.1042/bj20021321, PMID: 12429021 PMC1223209

[B31] Shannon P. Markiel A. Ozier O. Baliga N. S. Wang J. T. Ramage D. . (2003). Cytoscape: a software environment for integrated models of biomolecular interaction networks. Genome Res. 13, 2498–2504. doi: 10.1101/gr.1239303, PMID: 14597658 PMC403769

[B32] Tahir M. S. Tian L. (2021). HD2-type histone deacetylases: Unique regulators of plant development and stress responses. Plant Cell Rep. 40, 1603–1615. doi: 10.1007/s00299-021-02688-3, PMID: 34041586

[B33] Tian L. Chen Z. J. (2001). Blocking histone deacetylation in Arabidopsis induces pleiotropic effects on plant gene regulation and development. Proc. Natl. Acad. Sci. 98, 200–205. doi: 10.1073/pnas.98.1.200, PMID: 11134508 PMC14568

[B34] Voorrips R. (2002). MapChart: software for the graphical presentation of linkage maps and QTLs. J. heredity 93, 77–78. doi: 10.1093/jhered/93.1.77, PMID: 12011185

[B35] Wakeel A. Ali I. Khan A. R. Wu M. Upreti S. Liu D. . (2018). Involvement of histone acetylation and deacetylation in regulating auxin responses and associated phenotypic changes in plants. Plant Cell Rep. 37, 51–59. doi: 10.1007/s00299-017-2205-1, PMID: 28948334

[B36] Wang Z. Cao H. Chen F. Liu Y. (2014). The roles of histone acetylation in seed performance and plant development. Plant Physiol. Biochem. 84, 125–133. doi: 10.1016/j.plaphy.2014.09.010, PMID: 25270163

[B37] Wang H. Jiao X. Kong X. Liu Y. Chen X. Fang R. . (2020). The histone deacetylase HDA703 interacts with OsBZR1 to regulate rice brassinosteroid signaling, growth and heading date through repression of Ghd7 expression. Plant J. 104, 447–459. doi: 10.1111/tpj.14936, PMID: 33617099

[B38] Wang Y. Tang H. DeBarry J. D. Tan X. Li J. Wang X. . (2012). MCScanX: a toolkit for detection and evolutionary analysis of gene synteny and collinearity. Nucleic Acids Res. 40, e49–e49. doi: 10.1093/nar/gkr1293, PMID: 22217600 PMC3326336

[B39] Wu K. Zhang L. Zhou C. Yu C.-W. Chaikam V. (2008). HDA6 is required for jasmonate response, senescence and flowering in Arabidopsis. J. Exp. Bot. 59, 225–234. doi: 10.1093/jxb/erm300, PMID: 18212027

[B40] Xu Q. Liu Q. Chen Z. Yue Y. Liu Y. Zhao Y. . (2021). Histone deacetylases control lysine acetylation of ribosomal proteins in rice. Nucleic Acids Res. 49, 4613–4628. doi: 10.1093/nar/gkab244, PMID: 33836077 PMC8096213

[B41] Yang Z. Du J. Tan X. Zhang H. Li L. Li Y. . (2024). Histone deacetylase OsHDA706 orchestrates rice broad-spectrum antiviral immunity and is impeded by a viral effector. Cell Rep. 43, 113838. doi: 10.1016/j.celrep.2024.113838, PMID: 38386554

[B42] Yang X.-J. Seto E. (2007). HATs and HDACs: from structure, function and regulation to novel strategies for therapy and prevention. Oncogene. 26, 5310–5310. doi: 10.1038/sj.onc.1210599, PMID: 17694074

[B43] Yang J. Yuan L. Yen M. R. Zheng F. Ji R. Peng T. . (2020). SWI3B and HDA6 interact and are required for transposon silencing in Arabidopsis. Plant J. 102, 809–822. doi: 10.1111/tpj.14666, PMID: 31883159

[B44] Zhang R. Jia G. Diao X. (2023). geneHapR: an R package for gene haplotypic statistics and visualization. BMC Bioinf. 24, 1–13. doi: 10.1186/s12859-023-05318-9, PMID: 37189023 PMC10186671

[B45] Zhang J. Wu A. Wei H. Hao P. Zhang Q. Tian M. . (2020a). Genome-wide identification and expression patterns analysis of the RPD3/HDA1 gene family in cotton. BMC Genomics. 21, 1–16. doi: 10.1186/s12864-020-07069-w, PMID: 32948145 PMC7501681

[B46] Zhang K. Yu L. Pang X. Cao H. Si H. Zang J. . (2020b). In silico analysis of maize HDACs with an emphasis on their response to biotic and abiotic stresses. PeerJ 8, e8539. doi: 10.7717/peerj.8539, PMID: 32095360 PMC7023831

[B47] Zhao J. Li M. Gu D. Liu X. Zhang J. Wu K. . (2016). Involvement of rice histone deacetylase HDA705 in seed germination and in response to ABA and abiotic stresses. Biochem. Biophys. Res. Commun. 470, 439–444. doi: 10.1016/j.bbrc.2016.01.016, PMID: 26772883

