# Supplementary figures and images for: Genome-wide identification of the RPD3/HDA1 gene family in foxtail millet (Setaria italica) and analysis of their association with plant height
Source: Front Plant Sci. 2026 Jan 5;16:1722313. doi: 10.3389/fpls.2025.1722313 (PMC12812603; doi:10.3389/fpls.2025.1722313)

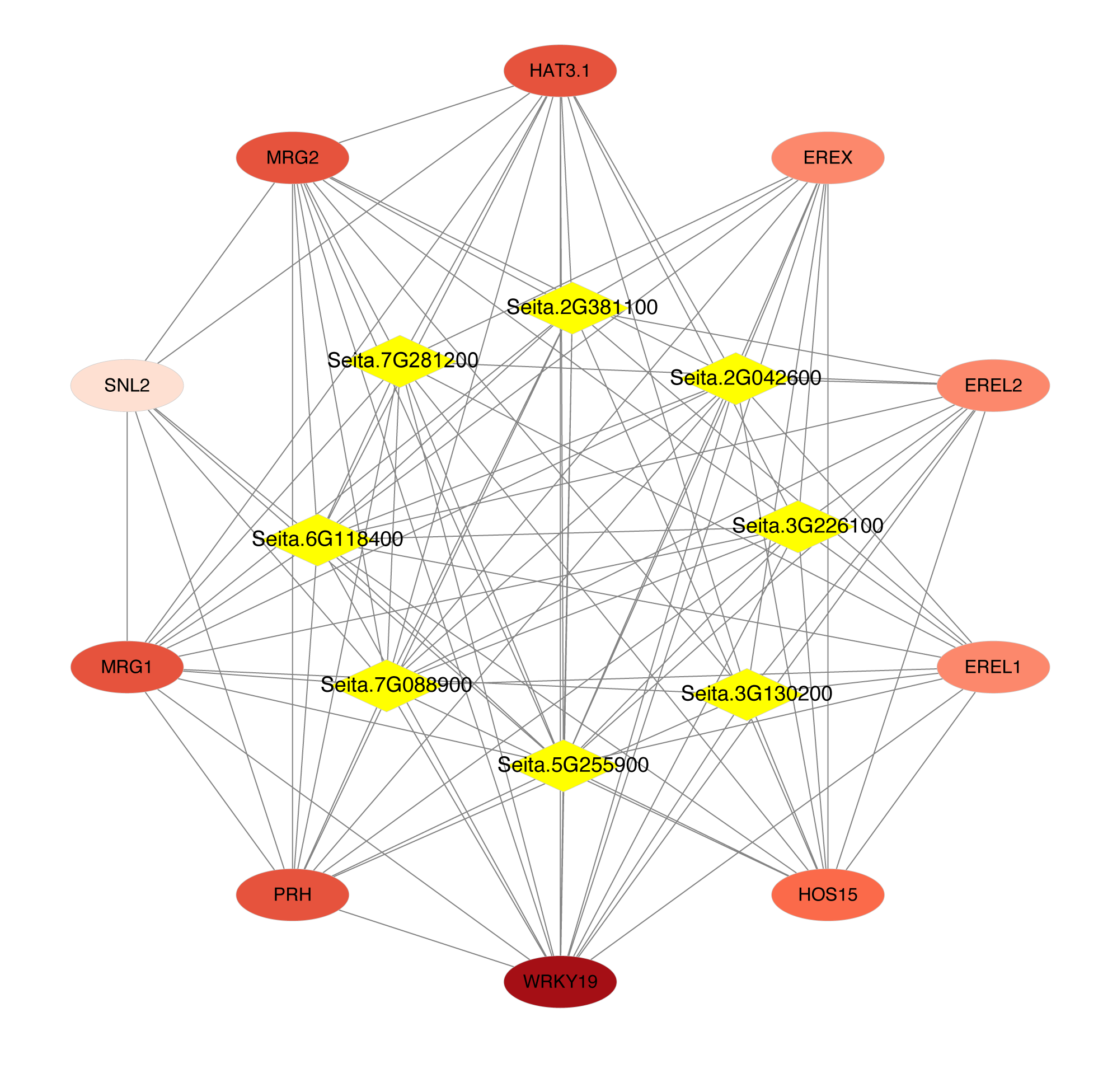

Supplement: Supplementary Figure 1 — SiPRD3/HDA1 homologous protein interaction network in Arabidopsis. [file Image1.tif]

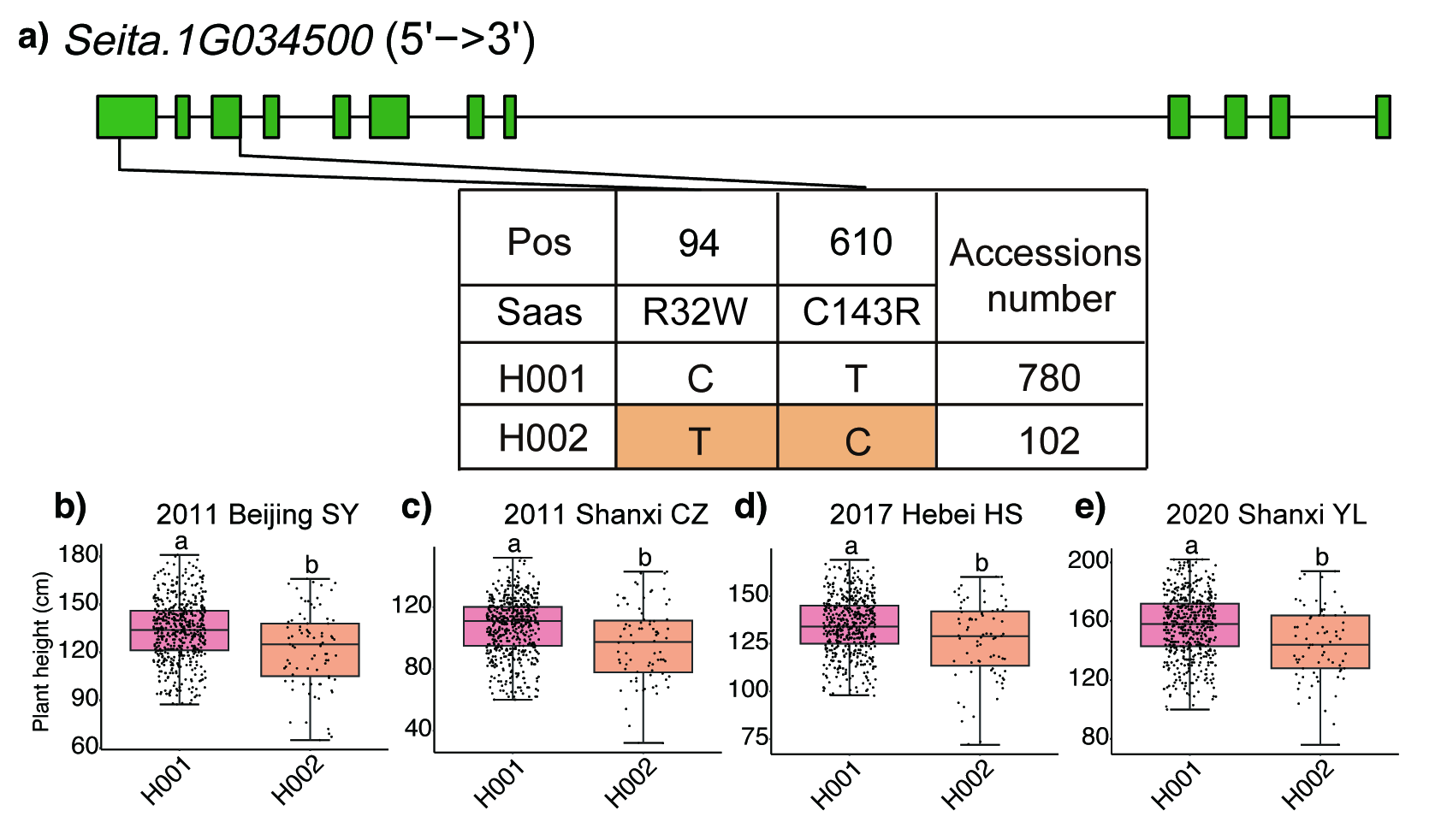

Supplement: Supplementary Figure 2 — Haplotype analysis of Seita.1G034500 in exons to assess effects on foxtail millet plant height [file Image2.tif]

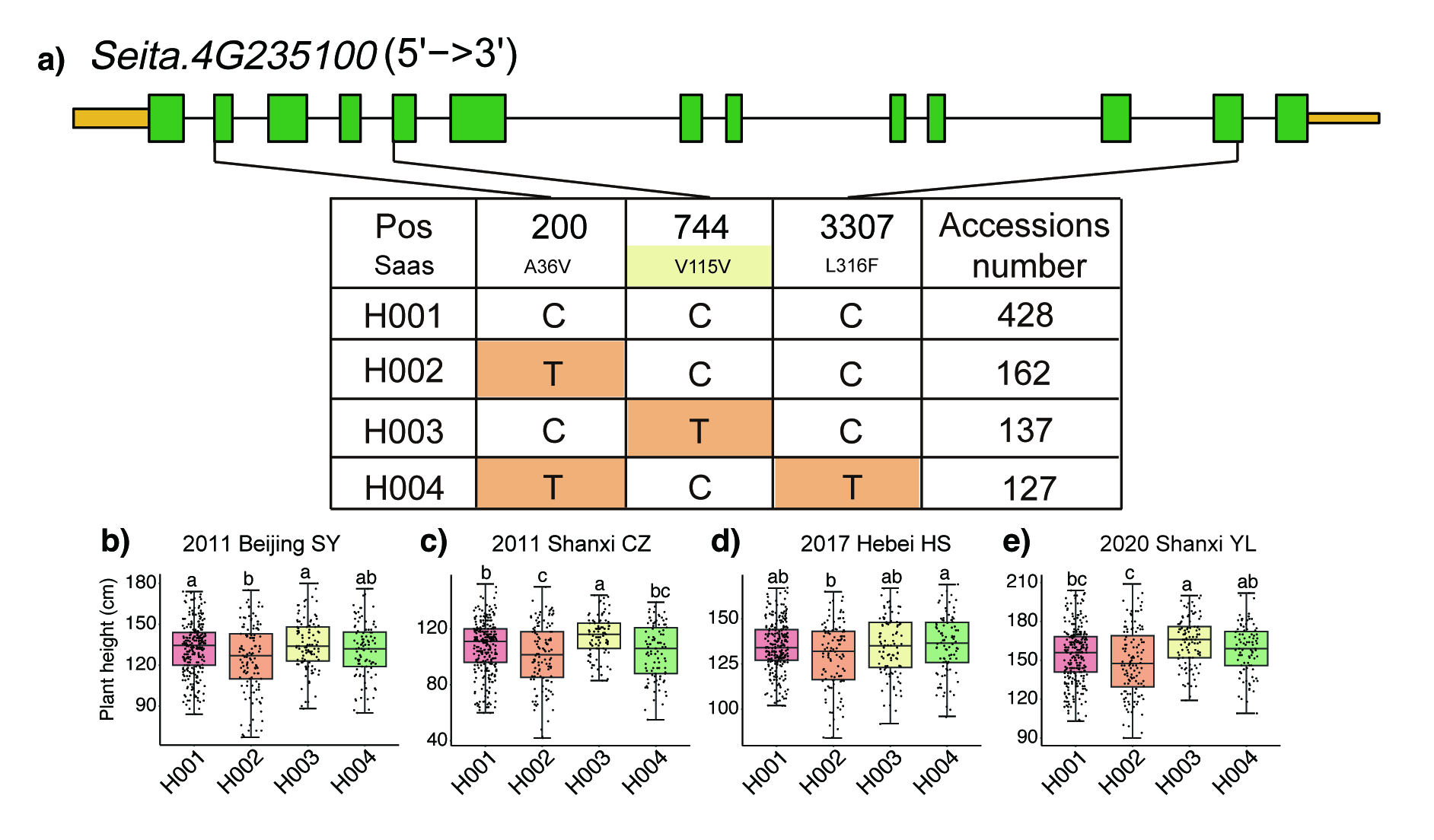

Supplement: Supplementary Figure 3 — Haplotype analysis of Seita.4G235100 in exons to assess effects on foxtail millet plant height. [file Image3.tif]

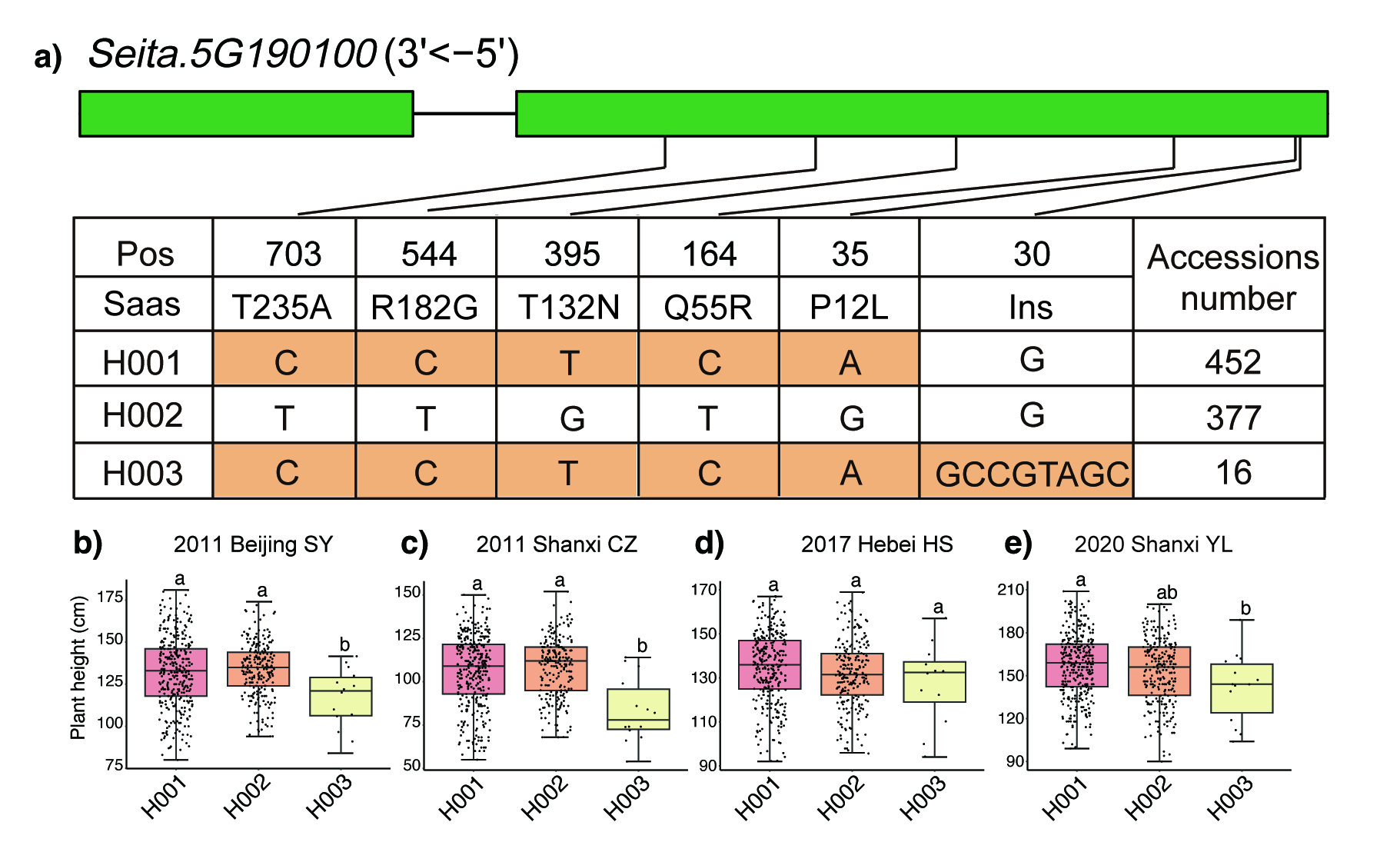

Supplement: Supplementary Figure 4 — Haplotype analysis of Seita.5G190100 in exons to assess effects on foxtail millet plant height. [file Image4.tif]

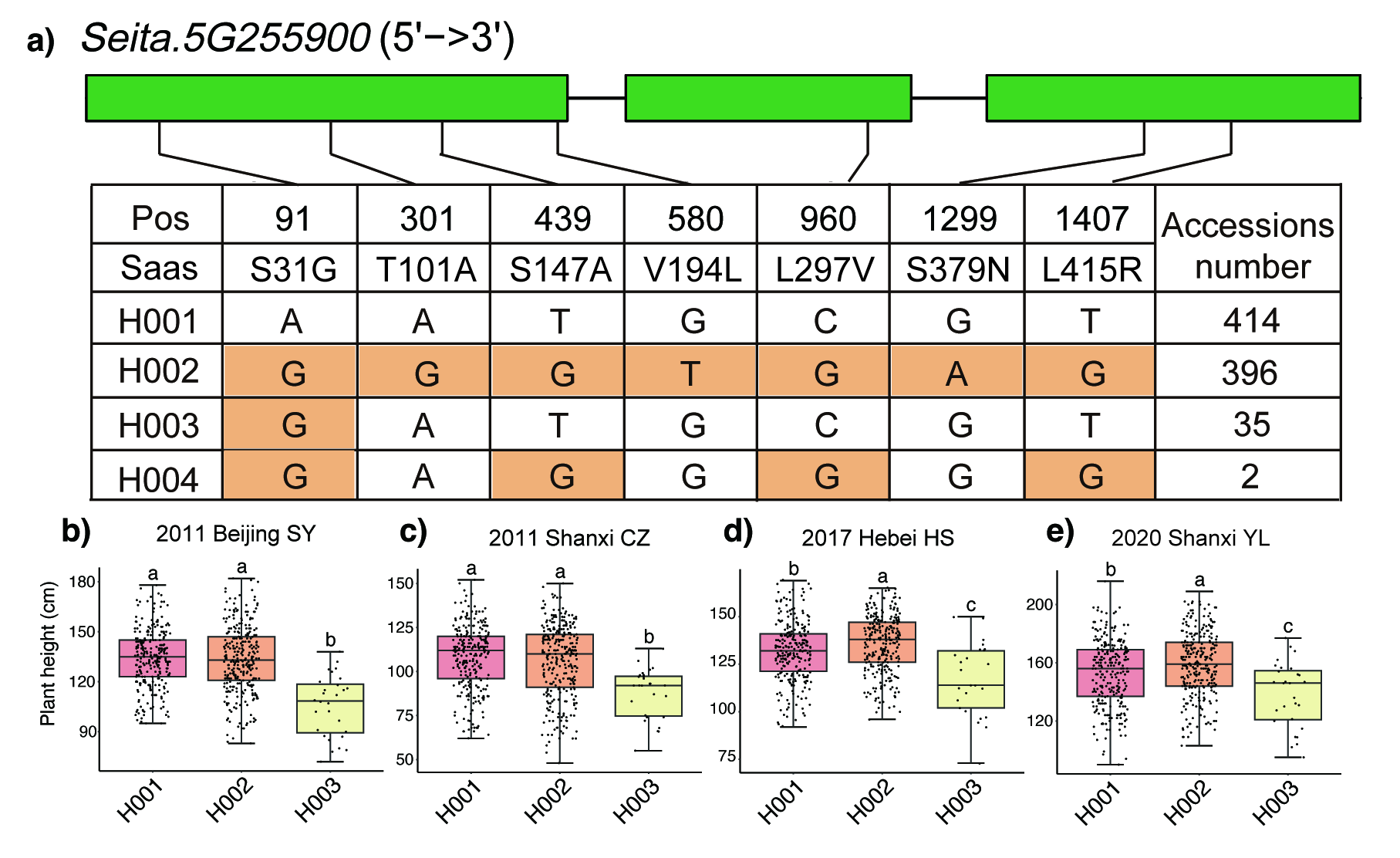

Supplement: Supplementary Figure 5 — Haplotype analysis of Seita.5G255900 in exons to assess effects on foxtail millet plant height. [file Image5.tif]

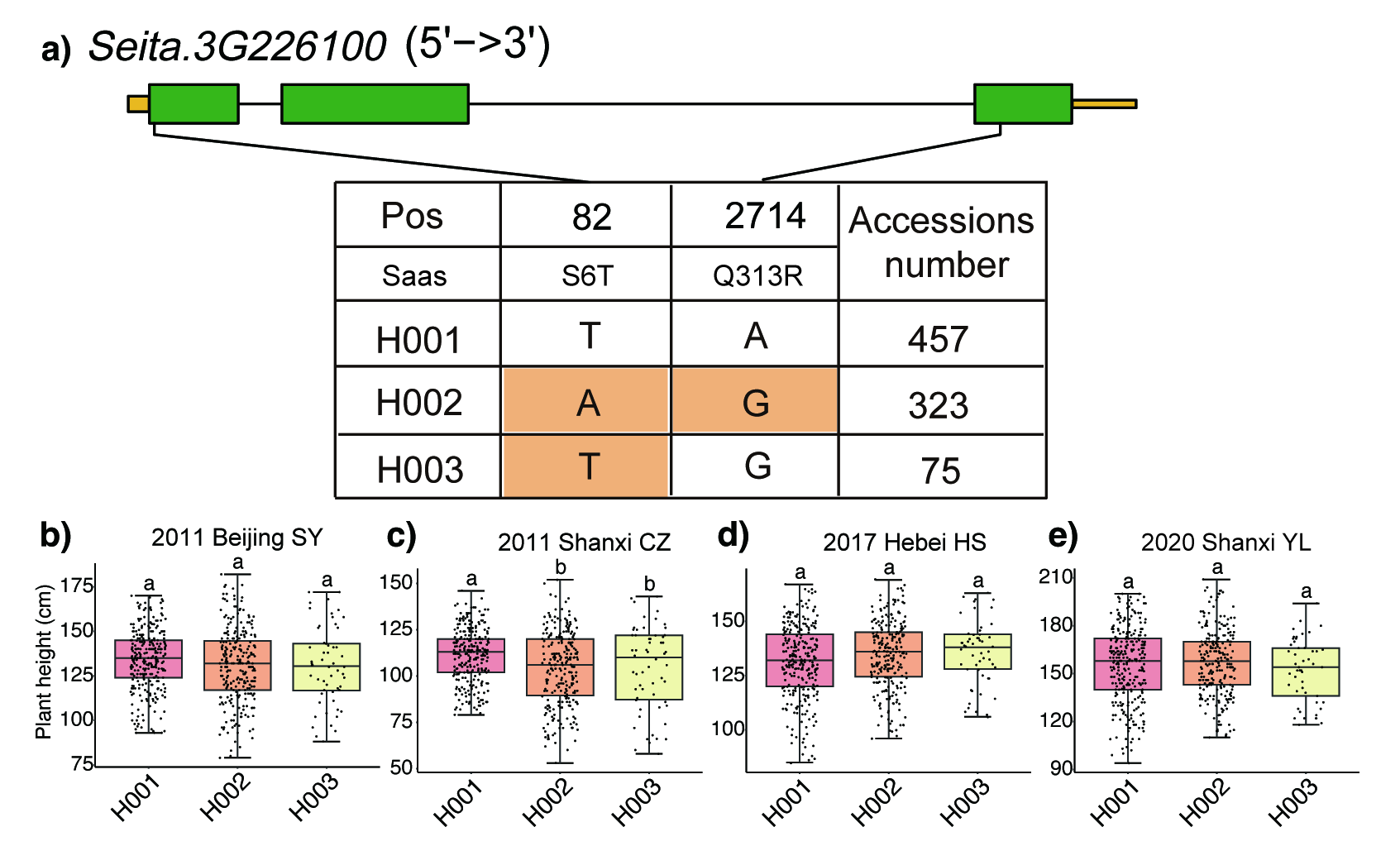

Supplement: Supplementary Figure 6 — Haplotype analysis of Seita.3G226100 in exons to assess effects on foxtail millet plant height. [file Image6.tif]

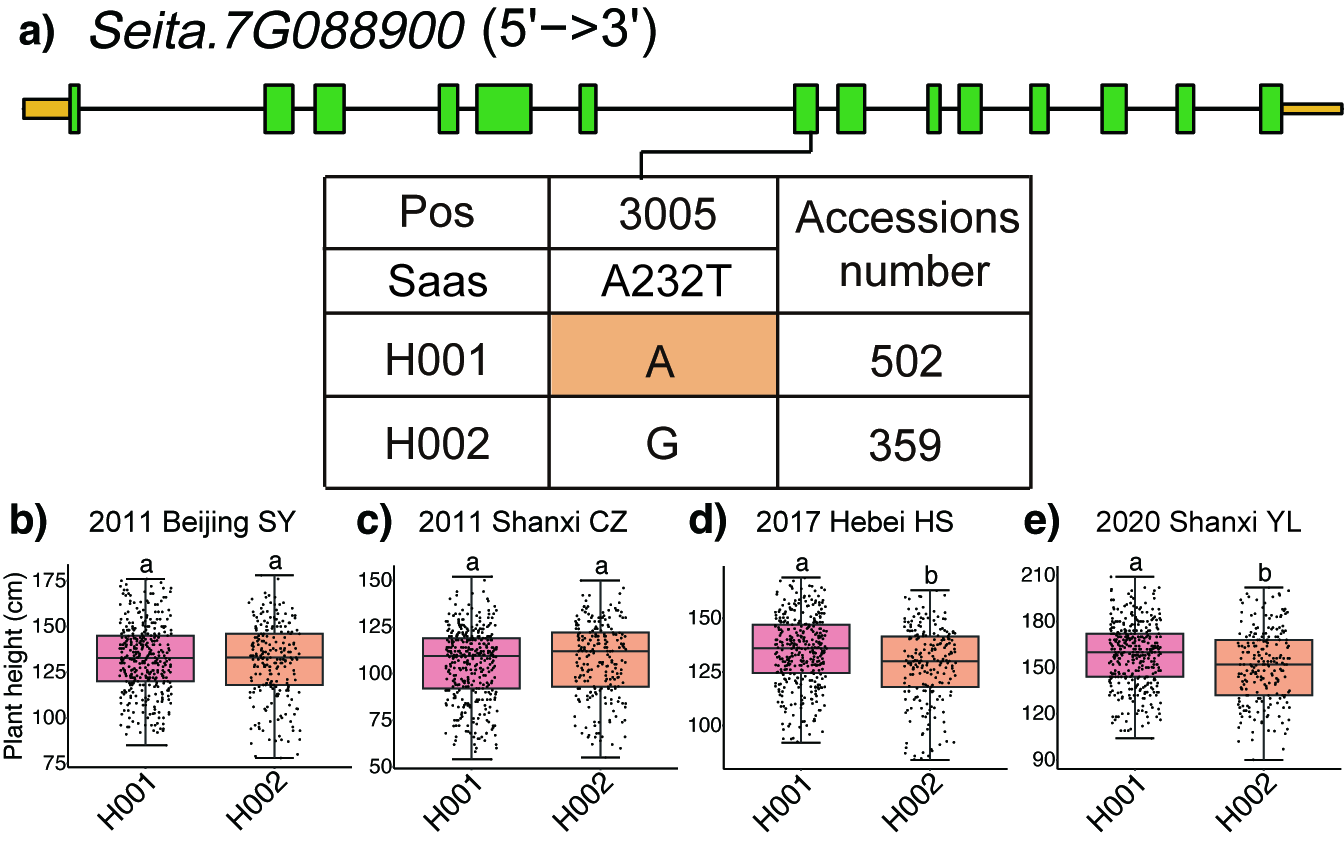

Supplement: Supplementary Figure 7 — Haplotype analysis of Seita.7G088900 in exons to assess effects on foxtail millet plant height. [file Image7.tif]

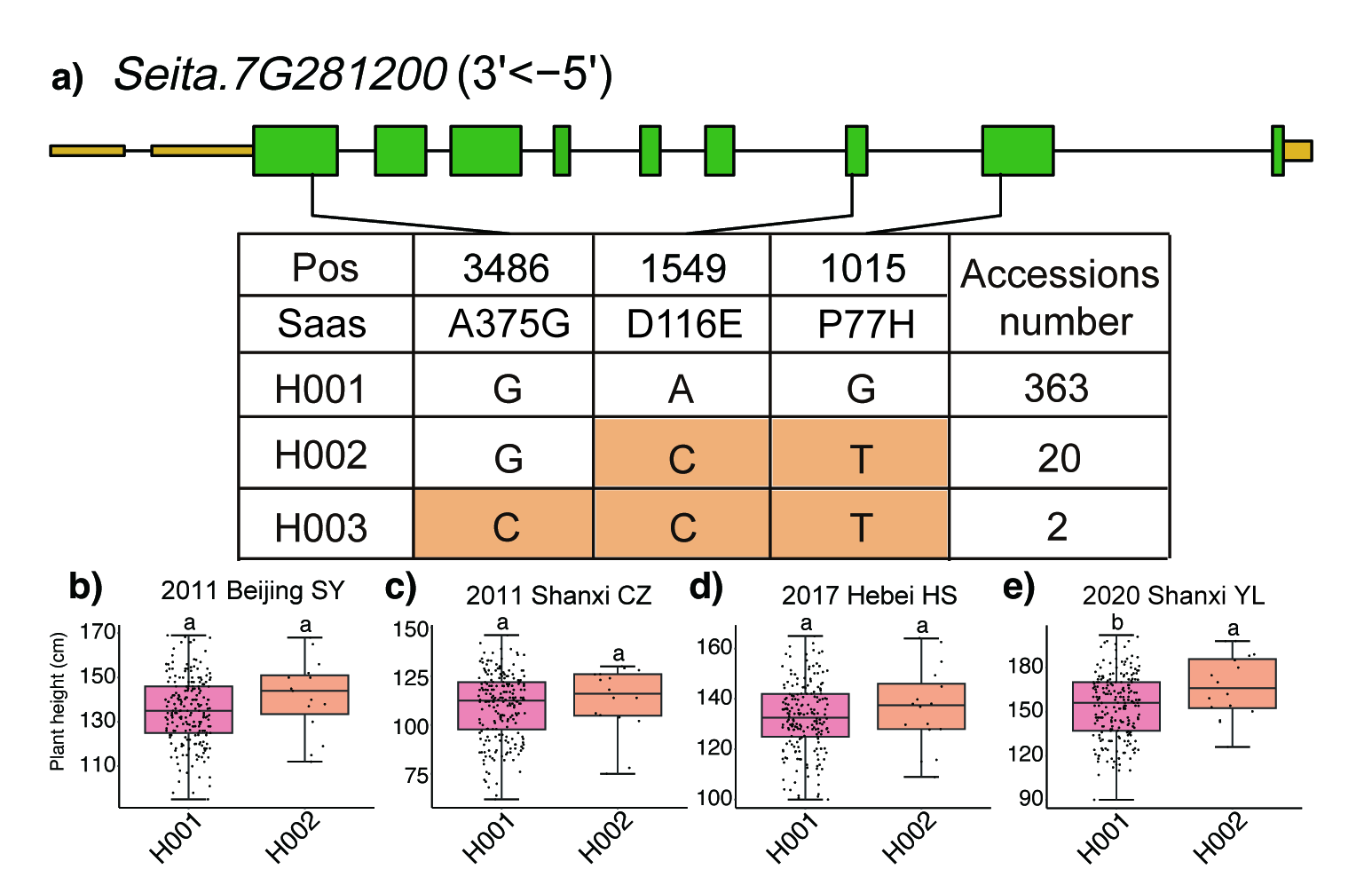

Supplement: Supplementary Figure 8 — Haplotype analysis of Seita.7G281200 in exons to assess effects on foxtail millet plant height. [file Image8.tif]

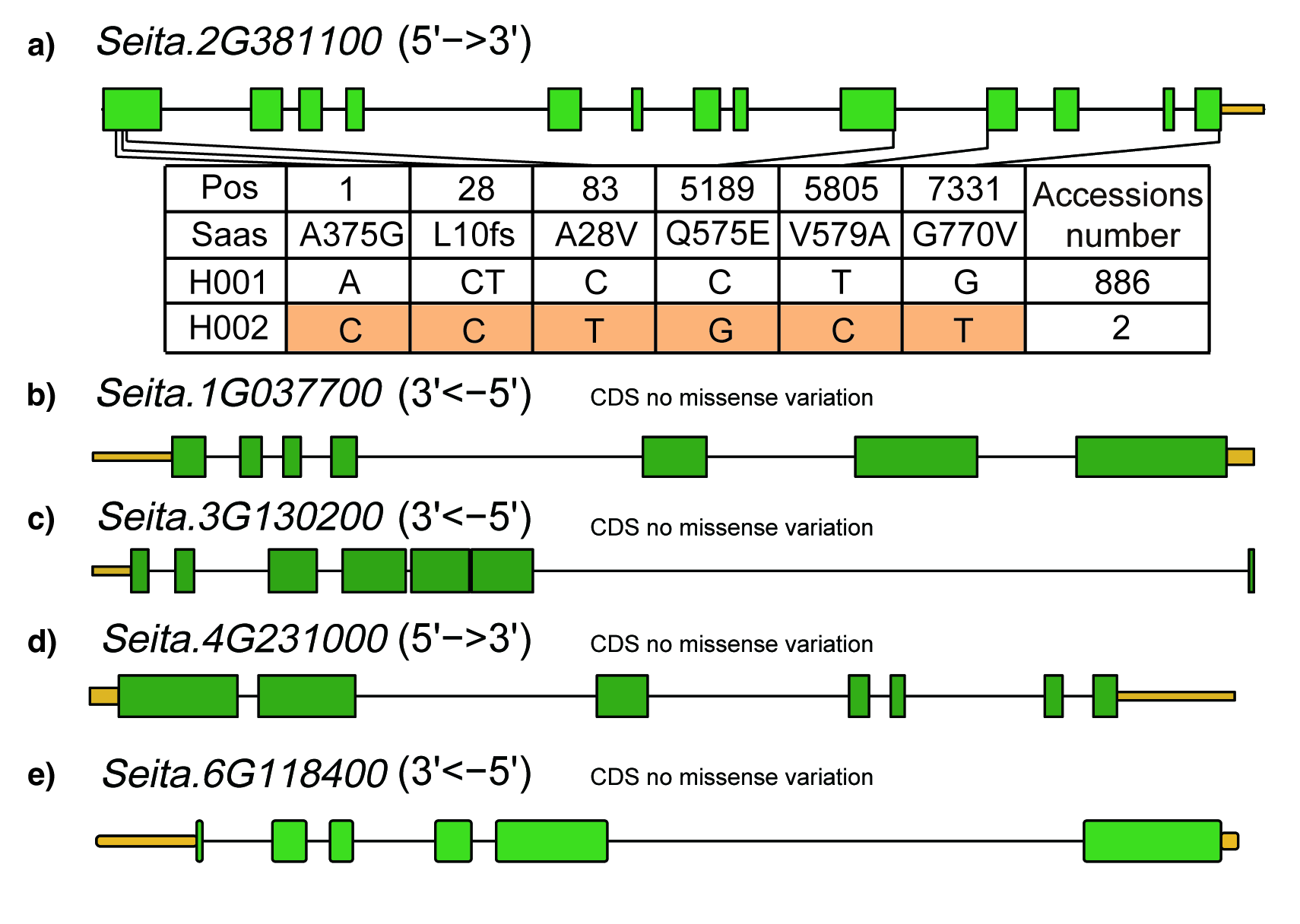

Supplement: Supplementary Figure 9 — Haplotype analysis of Seita.2G381100, Seita.1G037700, Seita.3G130200, Seita.4G231000 and Seita.6G118400 in exons to assess effects on foxtail millet plant height. [file Image9.tif]
